# Supplementary material for: Recapitulation of plaque formation, tau pathology, and neurodegeneration in a human 3D matrix model of Alzheimer’s disease
Source: Cell Rep Methods. 2026 Mar 30;6(4):101365. doi: 10.1016/j.crmeth.2026.101365 (PMC13106978; doi:10.1016/j.crmeth.2026.101365)
Supplement: Document S1. Figures S1–S6 [file mmc1.pdf]

Cell Reports Methods, Volume *6*

Supplemental information

Recapitulation of plaque formation, tau pathology, and neurodegeneration in a human 3D matrix model of Alzheimer's disease

Matthias Hebisch, Viola Kamin, Giovanna Cenini, Antonia Piazzesi, Fabio Bertan, Beatrice Weykopf, Julia Schlee, Senthilvelrajan Kaniyappan, Kevin J. Washicosky, Doo Yeon Kim, Daniele Bano, Michael Peitz, and Oliver Brüstle

Supplemental Figure 1

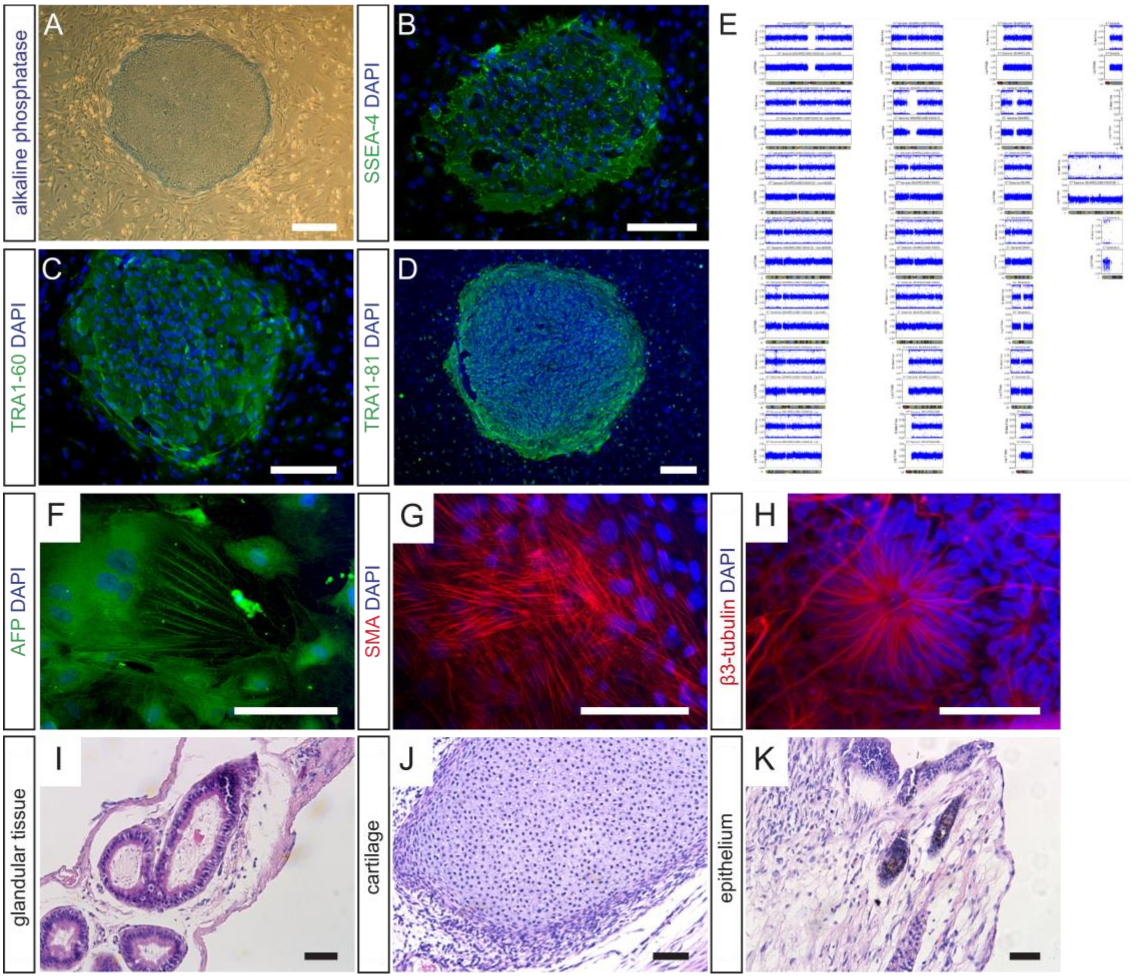

Parental iPS cell line, related to Figure 1

Expression of typical pluripotency markers in iPSC clone iLB-C14m-s11 was validated by immunohistochemistry for expression of (**A**) alkaline phosphatase, (**B**) SSEA4, (**C**) TRA-1-60 and (**D**) TRA-1-81. Scale bars = 100 µm. (**E**) Genomic integrity analysis of the iLB-C14m-s11 iPSC line via SNP panel. Differentiation potential was shown *in vitro* ((**F**) AFP (alpha-fetoprotein, marks endoderm), (**G**) SMA (smooth muscle actin, marks mesoderm), (**H**) β3-tubulin (marks ectoderm)) and *in vivo* via teratoma formation ((**I**) glandular tissue (endoderm), (**J**) cartilage (mesoderm) and (**K**) pigment epithelium (ectoderm)). Scale bar = 100 µm.

Supplemental Figure 2

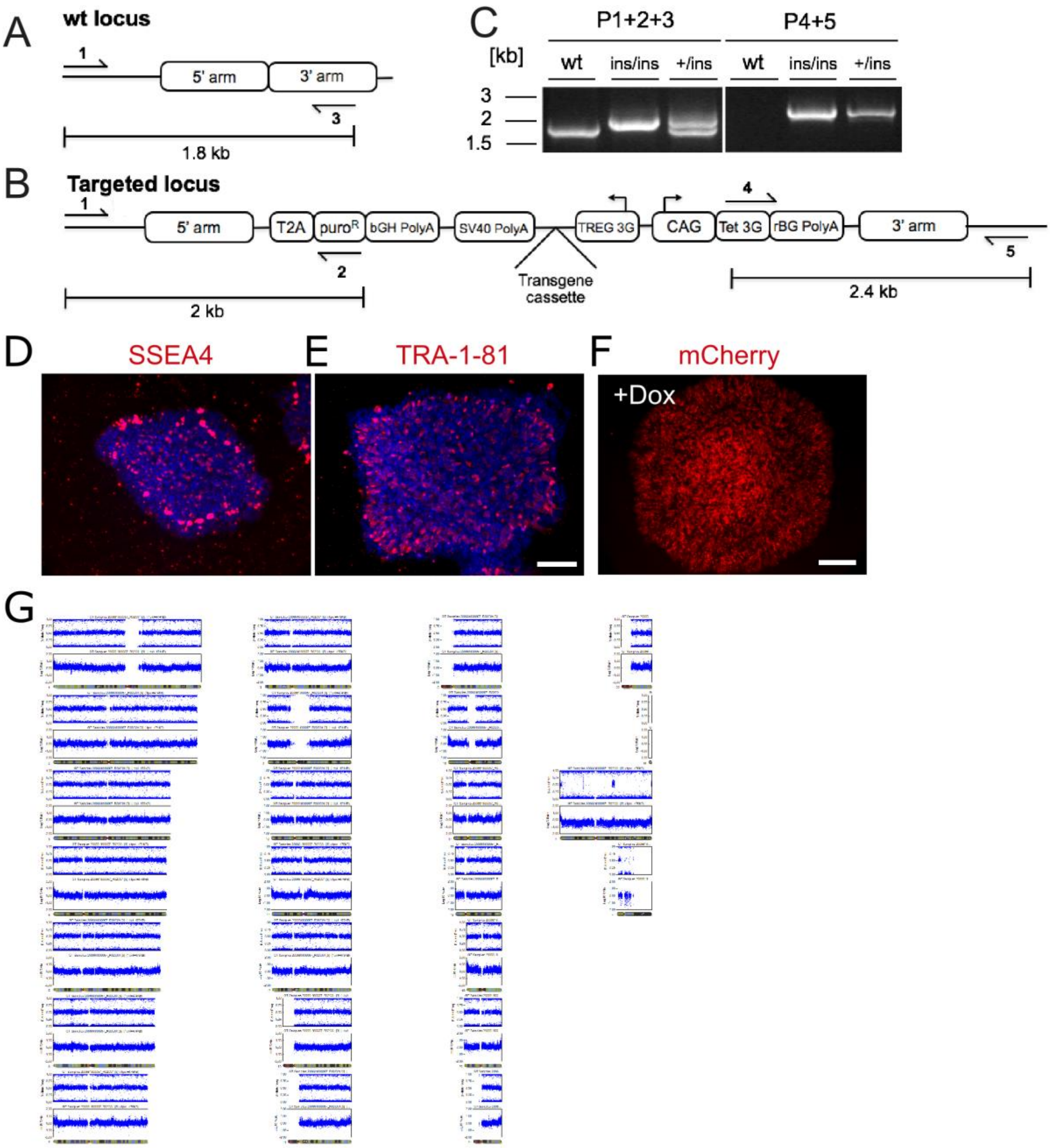

Validation of AAVS1 safe harbor transgene insertion, related to Figure 1

(**A**) Multiplex PCR using 3 primers for single-reaction analysis of 5‘ AAVS1 targeting in iPS colony samples of approximately 200-500 cells. Primer binding on untargeted alleles yields 1.8 kb products, whereas (**B**) binding outside the target region and inside the puromycin resistance gene yields a 2 kb product. A P4+5 pairing confirms the 3’ integration of the transgene cassette by generating a 2.4 kb product. (**C**) AAVS1-$APP_{Swe/Lon}$-*PSEN1ΔE9* genotyping in wild-type, homozygously and heterozygously integrated iPS cell clones. (**D,E**) Immunofluorescence detection of pluripotency-associated cell-surface

markers SSEA4 and TRA-1-81 (both red) in homozygous AAVS1-*APP*$_{Swe/Lon}$-*PSEN1ΔE9* iPS cell clones. Nuclear DAPI stain (blue). Scale bar = 100 µm. (**F**) Detection of the mCherry fluorescent marker in homozygous AAVS1-*APP*$_{Swe/Lon}$-*PSEN1ΔE9* iPS cells after 72 h of doxycycline induction. Scale bar = 200 µm. (**G**) Genomic integrity of homozygous AAVS1-APP$_{Swe/Lon}$-PSEN1ΔE9 iPS cells was assessed by SNP genotyping. For each chromosome the B allele frequency (upper row) and the log R ratio (lower row) are shown. No major genomic alterations could be detected.

Supplemental Figure 3

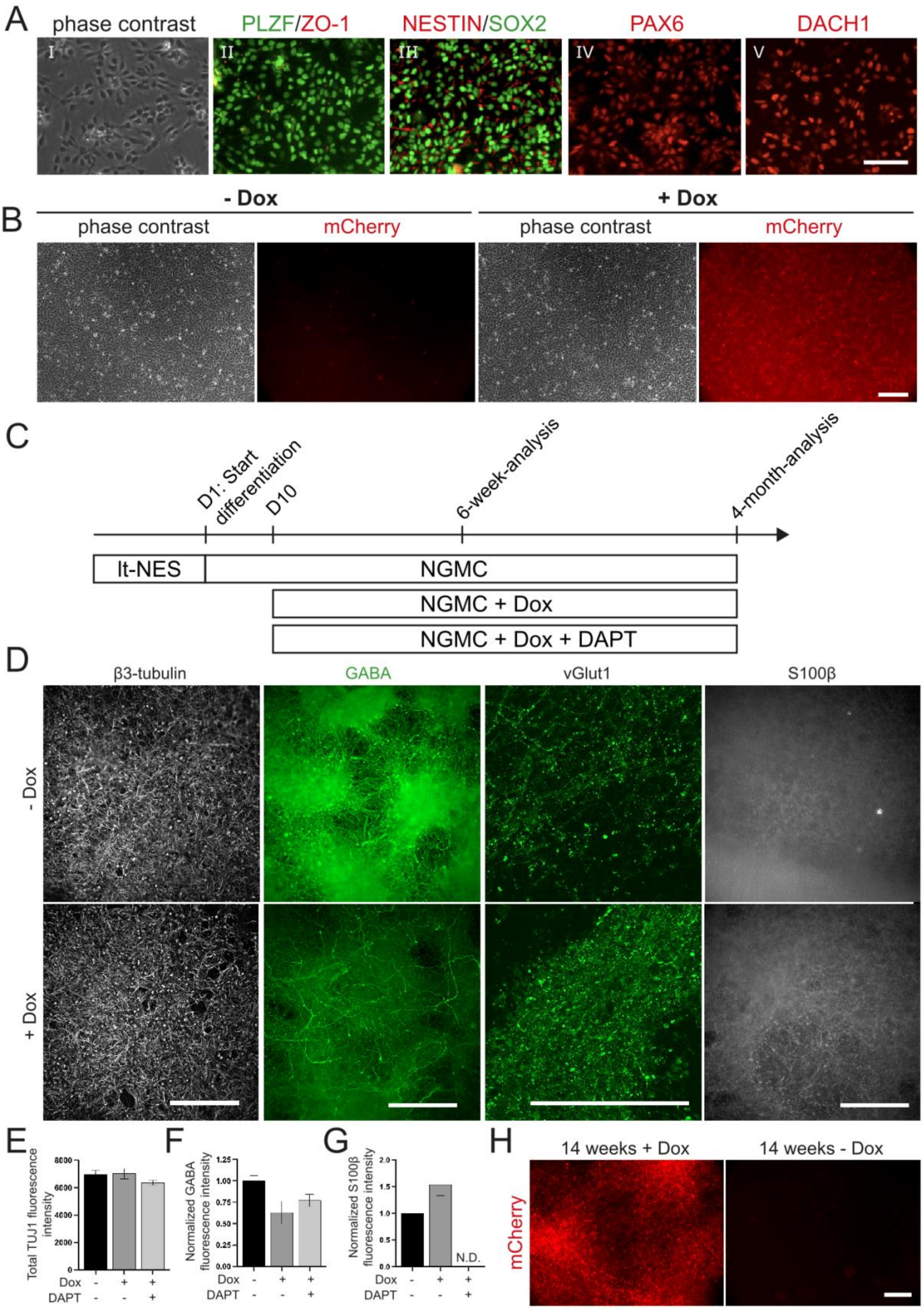

Validation of neural stem cells and neuronal cultures, related to Figure 1

(**A**) *$APP_{Swe/Lon\text{-}PSEN1\Delta E9}$* lt-NES cells exhibit a rosette-like growth pattern (phase contrast) and expression of the neural stem cell markers PLZF, ZO-1, NESTIN, SOX2, PAX6 and DACH1. Scale bar = 100 µm. (**B**) Dox-induced expression of the mCherry reporter in *$APP_{Swe/Lon\text{-}PSEN1\Delta E9}$* lt-NES cells. Scale bar = 200 µm. (**C**) Schematic experimental outline. Lt-NES cells are embedded in a 3D matrix and differentiated for 10 days prior to application of the experimental condition to avoid transgene interference with early differentiation. Cultures were analyzed after 6 weeks or 4 months of treatment. (**D**) Representative micrographs of high-density 3D homozygous *$APP_{Swe/Lon\text{-}PSEN1\Delta E9}$* neurons stained for β3-tubulin, GABA, vGlut1 and S100β after 6 weeks of growth-factor withdrawal with and without doxycycline treatment. Scale bars = 500 µm. (**E-G**) Quantification of neural markers β3-tubulin (E), GABA (F), astrocyte marker S100β (G) in *$APP_{Swe/Lon\text{-}PSEN1\Delta E9}$* lt-NES differentiated for 6 weeks ± Dox and DAPT treatment (n=3; Student's t-test, one-tailed, unpaired). (**H**) Validation of transgene induction in homozygous *$APP_{Swe/Lon\text{-}PSEN1\Delta E9}$* neurons after 14 weeks of differentiation in the presence and absence of doxycycline (1 µg/ml, mCherry in red). All images from high-density areas. Scale bar = 200 µm. All data presented as mean ± SEM.

Supplemental Figure 4

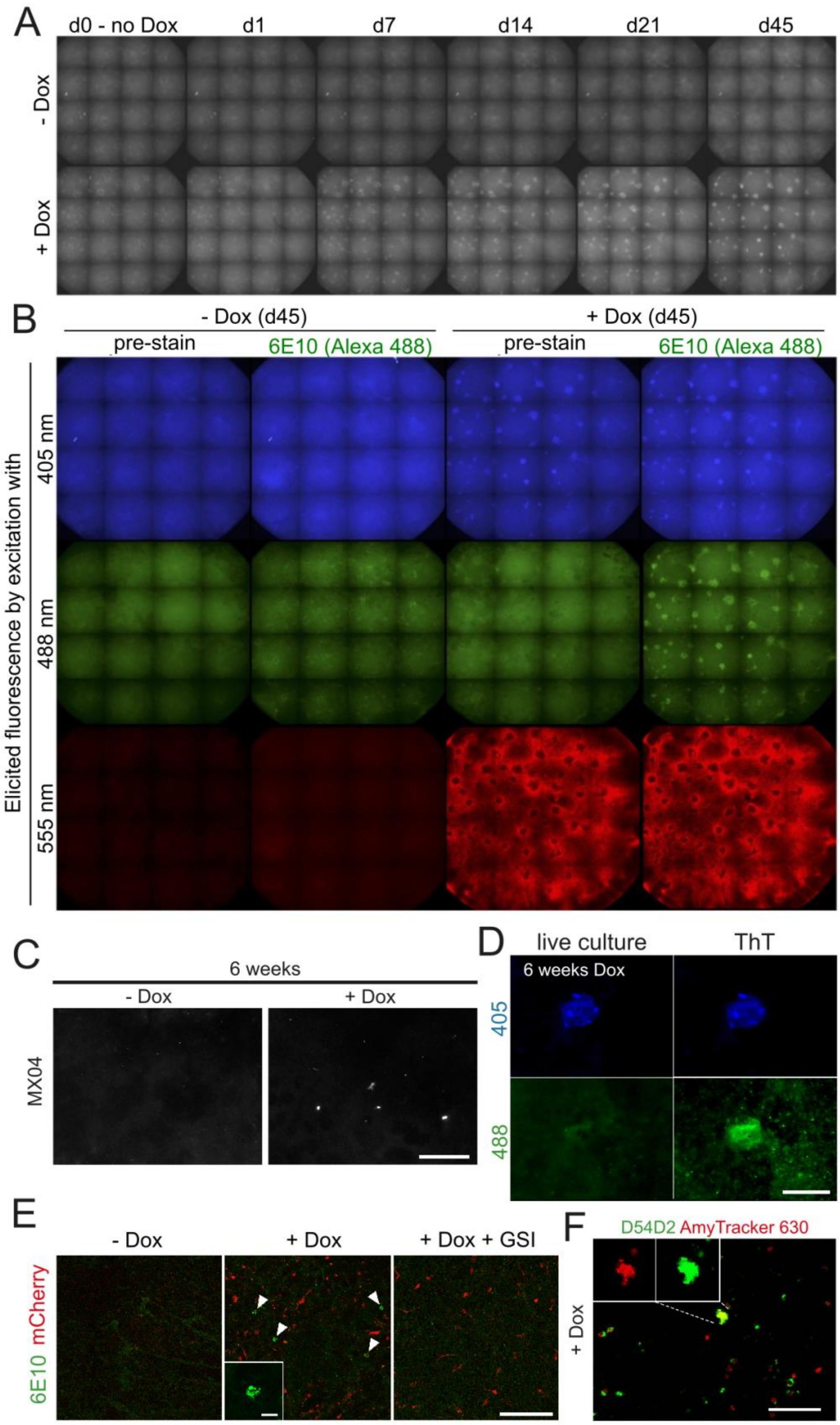

Autofluorescent amyloid deposits and dye-based stainings, related to Figure 2

(**A**) Stiched longitudinal overview of 96-well 3D cultures. Images show autofluorescence in the DAPI channel elicited by a 405 nm laser. (**B**) Stitched overview of 96-well 3D cultures from (A). Rows show fluorescence collected in the DAPI, GFP, and RFP channels upon excitation with 405 nm, 488 nm, and 555 nm, respectively. Pre-stain indicates fluorescence collected prior to antibody staining. 6E10 indicates fluorescence after antibody staining with 6E10 and secondary staining with an Alexa 488-tagged antibody. (**C**) Low magnification MX04 staining of 6-week-old cultures with and without doxycycline induction. MX04 fluorescence is depicted in greyscale (n=3). Scale bar = 200 µm. (**D**) Aggregate in a 6-week Dox-induced 3D culture. Autofluorescence elicited by 405 nm (emission in blue), and 488 nm (emission in green) light in a live culture and after PFA fixation and staining with ThT. Scale bar = 100 µm. (**E**) Detection of Aβ (6E10) and mCherry in 6-week Dox-induced, non-induced and induced GSI-treated 3D cultures. Confocal imaging; arrowheads indicate 6E10 accumulations. Scale bar = 100 µm, zoom-in: 10 µm. (**F**) Confocal micrograph of an amyloid aggregate with amyloid-β-specific antibody D54D2 (green) and amyloid fibril dye Amytracker 630 (red) in a 6-week Dox-induced culture. Scale bar = 50 µm.

Supplemental Figure 5

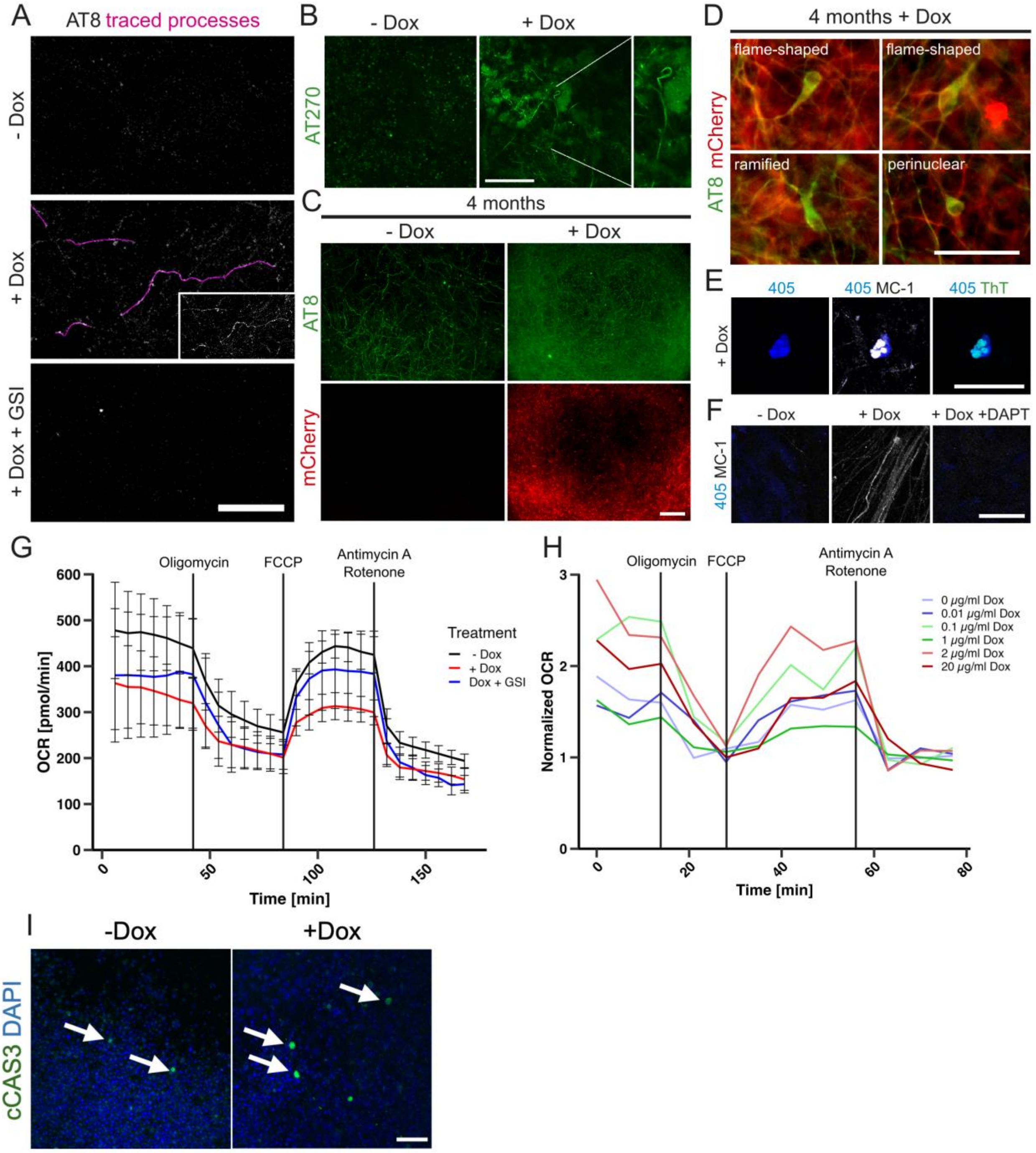

Tau pathology in *APP*$_{Swe/Lon}$, *PSEN1(ΔE9)* matrix cultures and assessment of mitochondrial respiration, related to Figures 3 and 4

(**A**) Illustration of traced AT8-positive neurites. Please note that the +Dox panel is also depicted as main Figure 3B. Scale bar = 100 µm. (**B**) 6-week Dox-induced cultures stained with the PHF antibody AT270. Magnification of a neuron with intraneuritic p-tau accumulation. Scale bar = 100 µm. (**C**) Example of a 4-month Dox-induced culture depicting anti-phospho-tau immunofluorescence (AT8, green) and mCherry (red). Scale bar = 200 µm. (**D**) High-magnification images of single 4-month Dox-induced neurons from (B)

showing p-tau accumulation and morphological alterations reminiscent of neurofibrillary tangles. Scale bar = 40 μm. (**E**) Aggregated p-tau in the "paperclip" conformation is a hallmark of tau pathology and neurofibrillary tangles. Detection of amyloid autofluorescence (blue), "paperclip" tau (MC-1 antibody, grayscale) and Thioflavin T (green) in 4-month Dox-induced cultures. Scale bar = 50 μm. (**F**) 4-months-induced cultures co-treated with 10 μM DAPT. Scale bar = 50 μm. (**G**) Oxygen consumption rate (OCR) of 6-week-induced lt-NES derived *APP*$_{Swe/Lon}$-*PSEN1ΔE9* neurons without induction (NGMC), treatment with Dox or Dox + the γ-secretase inhibitor DAPT (n=3; 6 technical replicates each). (**H**) Three weeks pre-differentiated, non-transgenic lt-NES-derived neurons were thawed and treated with 0.01 - 20 μg/ml doxycycline for 1 week before Seahorse measurement. Data were stratified into control (0), low (0.01, 0.1), medium (1, 2) and high (20) doxycycline concentrations for clarity (Lines represent mean values: 3 technical replicates per condition). (**I**) Cleaved caspase-3 staining of 6-week-induced 3D cultures with nuclear stain (DAPI) overlay. Single-plane confocal images. Arrows point at cCas3-positive cells. Scale bar = 100 μm.

Supplemental Figure 6

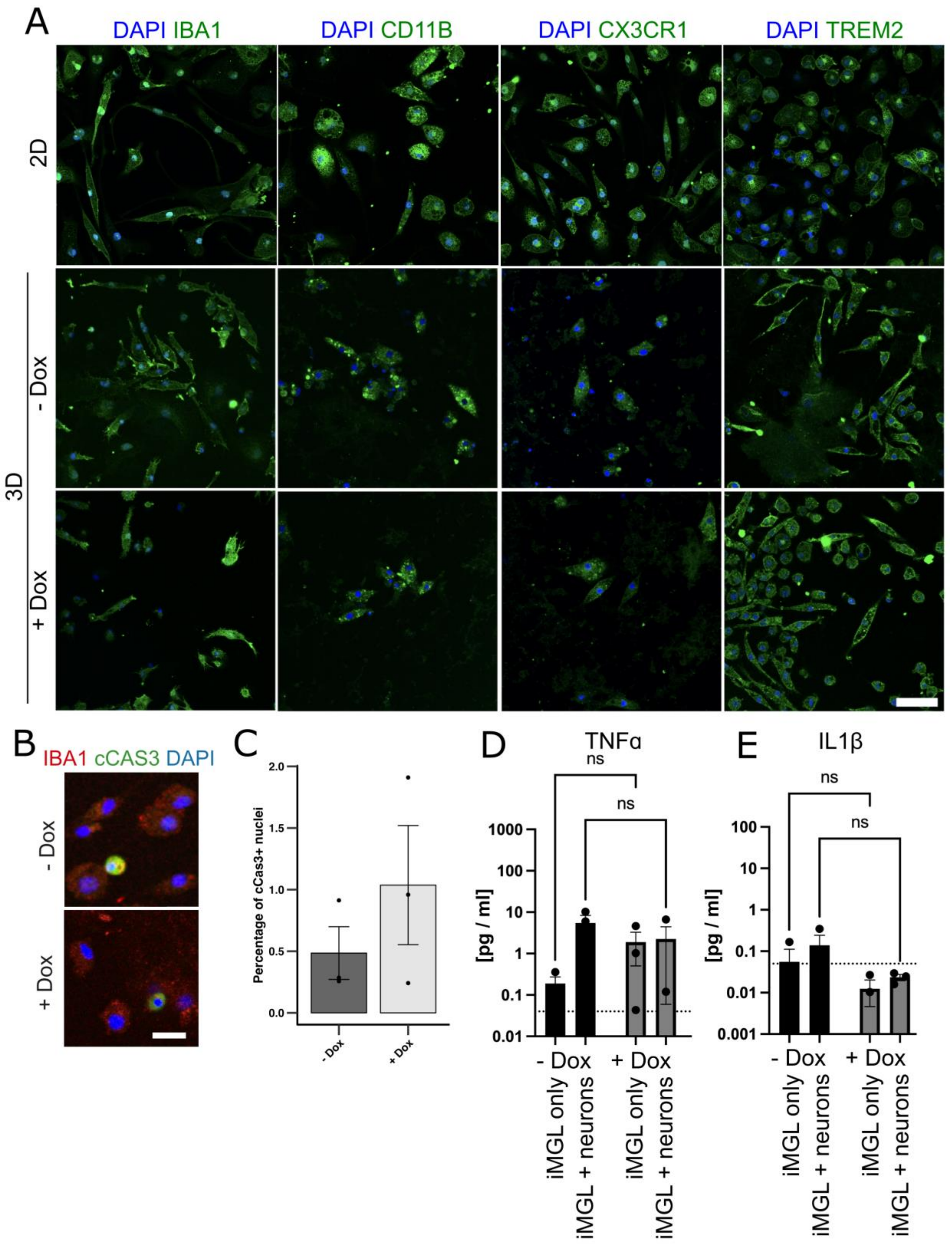

Human iPSC-derived microglia in monoculture in 2D and 3D settings, related to Figure 5

(**A**) Representative confocal images of microglial marker expression after 5 days in 2D or 3D culture. Scale bar = 50 µm. (**B**) Representative confocal images and (**C**) quantification of cCAS-positive cells in the

presence and absence of 1 µg/ml doxycycline (n=3). Scale bar = 25 µm. (**D**) MSD quantification of TNFα secretion into conditioned medium from 3D cultures of iPSdMiGs with or without a 5-day-co-culture with neurons. (**E**) MSD quantification of IL1β secretion into conditioned medium from 3D cultures of iPSdMiGs with or without a 5-day-co-culture with neurons. Kruskal-Wallis test with Nemenyi's post-hoc test. All data presented as mean ± SEM.
