# Supplementary material for: Recapitulation of plaque formation, tau pathology, and neurodegeneration in a human 3D matrix model of Alzheimer’s disease
Source: Cell Rep Methods. 2026 Mar 30;6(4):101365. doi: 10.1016/j.crmeth.2026.101365 (PMC13106978; doi:10.1016/j.crmeth.2026.101365)
Supplement: Document S2. Article plus supplemental information [file mmc2.pdf]

Cell Reports
Methods

Article

Recapitulation of plaque formation, tau pathology, and neurodegeneration in a human 3D matrix model of Alzheimer’s disease

Graphical abstract

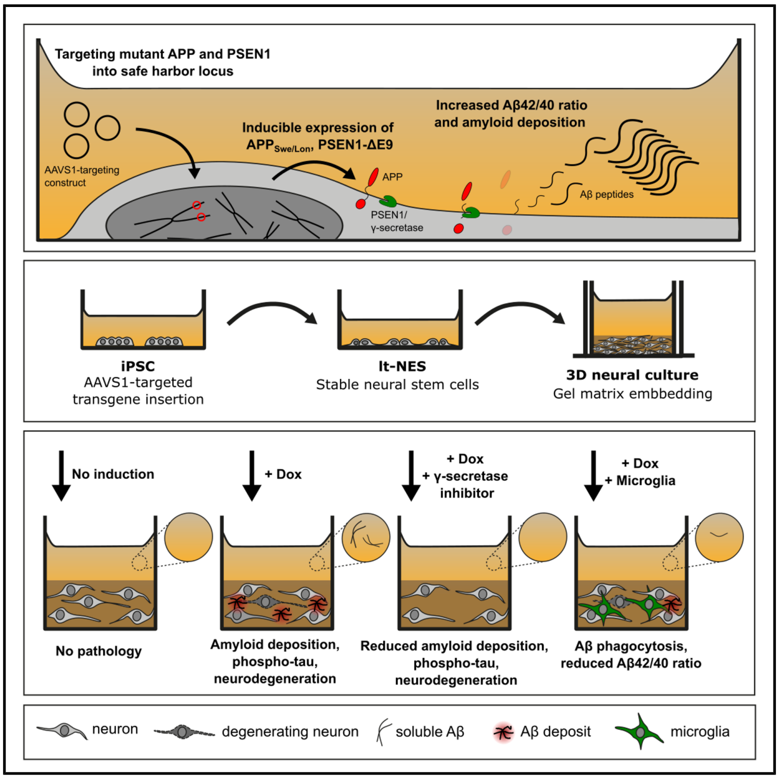

Authors

Matthias Hebisch, Viola Kamin, Giovanna Cenini, ..., Daniele Bano, Michael Peitz, Oliver Brüstle

Correspondence

brustle@uni-bonn.de

In brief

Hebisch et al. demonstrate that Alzheimer’s disease can be modeled in non-immortalized human neurons in 3-dimensional hydrogel cultures using only increased β-amyloid generation to drive other Alzheimer’s hallmarks like tau hyperphosphorylation, mitochondrial damage, and neuronal death. This model enables studies of late-stage Alzheimer’s mechanisms in human cells.

Highlights

- Genetically engineered iPSC-based 3D matrix model with pronounced Aβ deposition
- Recapitulates emerging tau pathology, mitochondrial dysfunction, and neurodegeneration
- Suitable for assessing Aβ-targeted rescue strategies
- Modular system suitable for microglia integration

Hebisch et al., 2026, Cell Reports Methods *6*, 101365
April 20, 2026 © 2026 The Author(s). Published by Elsevier Inc.
https://doi.org/10.1016/j.crmeth.2026.101365

CellPress

Cell Reports Methods

CellPress
OPEN ACCESS

Article

Recapitulation of plaque formation, tau pathology, and neurodegeneration in a human 3D matrix model of Alzheimer's disease

Matthias Hebisch,[1,2,7] Viola Kamin,[1,7] Giovanna Cenini,[1] Antonia Piazzesi,[3] Fabio Bertan,[3] Beatrice Weykopf,[1,4] Julia Schlee,[1] Senthilvelrajan Kaniyappan,[5] Kevin J. Washicosky,[2] Doo Yeon Kim,[2] Daniele Bano,[3] Michael Peitz,[1,6] and Oliver Brüstle[1,8,*]

[1]Institute of Reconstructive Neurobiology, University of Bonn Medical Faculty & University Hospital Bonn, 53127 Bonn, Germany
[2]Genetics and Aging Research Unit, MassGeneral Institute for Neurodegenerative Disease, Department of Neurology, McCance Center for Brain Health, Massachusetts General Hospital, Harvard Medical School, Charlestown, MA 02129, USA
[3]German Center for Neurodegenerative Diseases (DZNE), 53127 Bonn, Germany
[4]Cellomics Department, LIFE & BRAIN GmbH, 53127 Bonn, Germany
[5]Pharma Research and Early Development, Roche Innovation Center Basel, F. Hoffmann-La Roche Ltd., 4070 Basel, Switzerland
[6]Cell Programming Core Facility, University of Bonn, 53127 Bonn, Germany
[7]These authors contributed equally
[8]Lead contact
*Correspondence: brustle@uni-bonn.de
https://doi.org/10.1016/j.crmeth.2026.101365

MOTIVATION Modeling Alzheimer's disease (AD) comprehensively *in vitro* has been a long-standing challenge due to difficulties in recapitulating key disease hallmarks in a mechanistically connected manner. While previously used immortalized cell models captured aspects of plaque formation and tau pathology in 3D, overexpression of immortalizing oncogenes restricts the study of neurodegenerative processes. Here, we set out to develop an *in vitro* AD model based on non-transformed human iPSC-derived neural cells.

SUMMARY

This study aims at implementing a 3D cell culture model of Alzheimer's disease (AD). To that end we engineered human induced pluripotent stem cell (iPSC)-derived neural stem cells to conditionally overexpress FAD mutant *APP* and *PSEN1* variants. After differentiation in 3D basement membrane matrices, cultures exhibited increased $A\beta_{42}$ and $A\beta_{40}$ levels and a highly pathogenic shift of the $A\beta_{42/40}$ ratio. Typical AD phenotypes such as amyloid deposition and tau pathology were observed alongside impaired mitochondrial integrity and neuronal damage. Pathophenotypes were ameliorated by γ-secretase inhibition, confirming amyloid toxicity as main driver of AD pathology. iPSC-derived microglia added to the cultures engulfed Aβ and apoptotic cells, underscoring the modularity of this experimental system. We expect our model to provide a useful tool for assessing the impact of amyloid reduction on downstream AD pathologies such as mitochondrial dysfunction, neuroinflammation, and neurodegeneration, in particular in light of recent progress in the development and use of amyloid-targeting drugs.

INTRODUCTION

Alzheimer's disease (AD) is the most prevalent neurodegenerative disorder and leading cause of dementia in the aged population worldwide.[1,2] It represents a major challenge for basic research and drug development, especially since there are only few models with low-grade pathology based on non-transformed authentic human neurons.[3–7] The last decade has seen the development of numerous AD-like *in vitro* models based on induced pluripotent stem cell (iPSC) technology in classical 2D cultures; a thorough overview of available iPSC-based models and their reported phenotypes has been compiled by the Tsai lab.[8,9] A lot of ground has been covered studying the toxic effects of β-amyloid (Aβ) and hyperphosphorylated tau (p-tau) in sporadic AD, familial AD (FAD), and Down syndrome (DS) using iPSC-derived neurons, astrocytes, oligodendrocytes, microglia, and pericytes. AD-related mutations can cause oxidative stress, cytoskeletal changes, impaired endo-/lysosomal trafficking and processing, altered cytokine release and calcium homeostasis, reduced synapse count, increased cholesterol content in astrocytes, DNA damage, and apoptosis. However, most models typically show somewhat mild phenotypes and lack key features

This is an open access article under the CC BY license (http://creativecommons.org/licenses/by/4.0/).

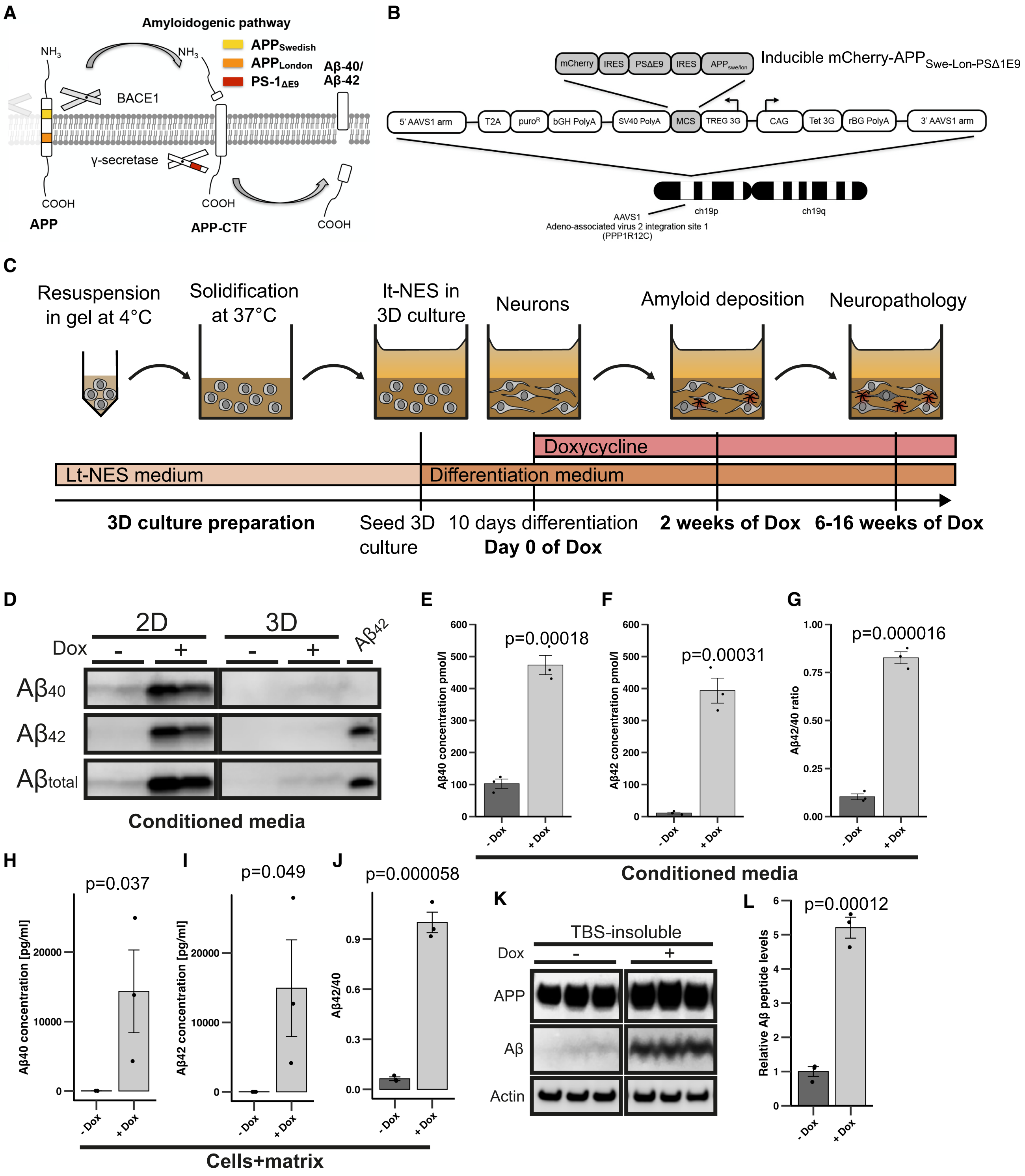

Figure 1. Generation and deposition of amyloid-β in 3D cultures

(A) Experimental concept: *APP* carrying the Swedish and London mutations (*APP*$_{Swe/Lon}$) is sequentially processed by β- (BACE1) and γ-secretase (containing the *PSEN1(ΔE9)* mutation), leading to elevated Aβ40 and Aβ42 release.

(B) Targeted insertion of the *APP*$_{Swe/Lon}$-*PSEN1(ΔE9)*-mCherry construct into the mammalian AAVS1 safe-harbor locus.

(C) Schematic of 3D culture generation, pre-differentiation, and induction of transgene expression.

(legend continued on next page)

of AD, such as extracellular deposition of amyloid, neurofibrillary tangle formation and neuroinflammatory interactions—likely due to only moderately elevated Aβ levels. 3D systems such as cerebral organoids of sporadic AD cases and chimeric transplants come with their own challenges such as increased complexity and limited scalability.[5–7,10–12] We and others have previously established 3D models overexpressing the FAD mutations *APP*$_{Swe/Lon}$ and *PSEN1(ΔE9)* to generate substantially higher levels of Aβ and achieve robust extracellular enrichment, so that amyloid deposition and toxicity are readily apparent.[13,14] These models also offer a high degree of modularity to introduce various cell types, such as microglia.[15] However, the currently available 3D models are based on a v-Myc-immortalized human neural progenitor line (ReN cell VM). Chronic expression of v-Myc is likely to modulate AD-specific pathological pathways as v-Myc is an oncogene and master transcriptional regulator of up to 15% of active human genes including pathways for cell metabolism, ATP generation, and apoptosis.[16] An attractive alternative to such an oncogene-based cell system for *in vitro* modeling of AD could combine the aggregation-promoting properties of FAD gene overexpression in 3D matrix arrays with the authenticity provided by non-transformed iPSC-derived neural cells. However, constitutive overexpression of FAD mutations may skew developmental trajectories and prime the model for pathology in human and mouse models of AD.[17,18] Thus, it is desirable to control transgene expression in order to commence the pathogenic cascade after neurodifferentiation is complete. In this study, we describe such a standardized, modular 3D culture model of AD that combines authentic neural cells, inducible overexpression of FAD mutants, robust and controllable AD-associated pathology, and amenability to the inclusion of human iPSC-derived microglia.

RESULTS

APP$_{Swe/Lon\text{-}PSEN1\Delta E9}$ *mutant* lt-NES cells show unimpaired differentiation in 3D cultures

In order to establish an *in vitro* model of AD that recapitulates the major pathological hallmarks of AD in non-transformed authentic human neurons, we inserted a doxycycline (Dox)-inducible transgene cassette consisting of *APP*$_{Swe/Lon}$, *PSEN1(ΔE9)*, and an mCherry reporter into a targeting plasmid for the genomic "safe-harbor" AAVS1 (Figure 1A).[13,19] The targeting plasmid contains a puromycin selection cassette and the Tet-On transactivator system to provide an all-in-one donor vector for single-step targeting of the human AAVS1 safe-harbor locus on chromosome 19 (AAVS1-*APP*$_{Swe/Lon\text{-}PSEN1\Delta E9}$ plasmid; Figure 1B).[20] iPSCs from a healthy subject (Figure S1) were nucleofected with the AAVS1-*APP*$_{Swe/Lon\text{-}PSEN1\Delta E9}$ plasmid. Inserted clones were validated for homozygous cassette integration by PCR genotyping of the AAVS1 locus and tested for construct inducibility and pluripotency marker expression (SSEA4, TRA-1-60), as well as genomic integrity via SNP analysis (Figures S2A–S2G). Validated clones were differentiated into lt-NES cells to provide a stable intermediary population for standardized neural differentiation. Lt-NES robustly generate a mix of neurons and astrocytes with a regional identity near the mid-hind brain boundary, which is similar to the regionalization of ReN VM cell-derived neurons in earlier AD models.[21,22] The cells were checked for expression of the neural stem cell markers PLZF, ZO-1, NESTIN, SOX2, PAX6, and DACH1 (Figure S3A) and construct inducibility (Figure S3B).[21,23] Choi and colleagues established separate 3D culture systems for cellular imaging ("thin-layer" cultures in imageable microwell plates) and protein analysis ("thick-layer" cultures in transwell plates allowing for a larger medium supply) that were used here in the same fashion.[13] For neural differentiation, *APP*$_{Swe/Lon\text{-}PSEN1\Delta E9}$ lt-NES cells were first embedded in a 3D matrix, and then subjected to growth factor withdrawal to induce differentiation. After 10 days, the respective experimental condition (either NGM medium only, NGM + Dox or NGM + Dox + γ-secretase inhibitor (GSI)) was applied for the remainder of the experiment (Figures 1C and S3C). Upon differentiation, the cultures developed a dense network of β3-tubulin-positive neurons. After 4 weeks, both vGLUT1 and GABA-positive neurons as well as S100β-positive astrocytes were detected, which is in line with previous data on differentiated lt-NES cells.[21,22] No obvious differences were found between non-induced and transgene-induced cultures as assessed by fluorescence intensity measurements (Figures S3D–S3G).[24] Thus, triple mutant *APP*$_{Swe/Lon\text{-}PSEN1\Delta E9}$ lt-NES cells cultured in our 3D matrix system show no impaired differentiation.

Transgene induction results in robust Aβ accumulation within the matrix

Attempts to model AD *in vitro* using *APP* overexpression have failed to produce amyloid plaques due to excessive washout of Aβ during medium changes.[25–27] 3D cultivation avoids this problem as Aβ is retained in the 3D matrix instead of entering the culture supernatant.[13,14,25,26] To validate Aβ retention in the gel matrix, supernatants from 4-week-treated 2D monolayer and 3D cultures were compared in western blot analysis. In the supernatants from 2D cultures, low baseline Aβ secretion was drastically boosted after Dox induction for 4 weeks, indicating Aβ washout (Figure 1D, left column). In contrast, 3D culture-derived supernatants showed barely any Aβ under

(D) Detection of Aβ_{40}, Aβ_{42}, and total Aβ in supernatants of *APP*$_{Swe/Lon\text{-}PSEN1\Delta E9}$ 2D and 3D cultures after 4 weeks of Dox-induction (2×10^6 cells per well in 2 mL medium, 48 h after media change; $n = 2$).

(E and F) ELISA measurements of Aβ_{40} and Aβ_{42} in *APP*$_{Swe/Lon\text{-}PSEN1\Delta E9}$ 2D cultures after 6 weeks of Dox induction (2×10^6 cells per well in 2 mL medium; 24 h after media change; $n = 3$). One-tailed Student's t test (unpaired).

(G) Aβ42/40 ratio calculated from sample pairs. One-tailed Student's t test (unpaired). Values are presented as mean ± SEM.

(H and I) MSD ELISA measurements of Aβ_{40} and Aβ_{42} in *APP*$_{Swe/Lon\text{-}PSEN1\Delta E9}$ 3D cultures after 6 weeks of Dox induction (2 mL medium, 24 h after media change; $n = 3$). One-tailed Student's t test (unpaired).

(J) Aβ42/40 ratio calculated from sample pairs. One-tailed Student's t test (unpaired).

(K) Western blot analysis and (L) quantification of in *APP*$_{Swe/Lon\text{-}PSEN1\Delta E9}$ thick-layer 3D cultures after 4 months of Dox induction. Data presented as mean ± SEM; $n = 3$; one-tailed Student's t test; unpaired. See also Figures S1–S3.

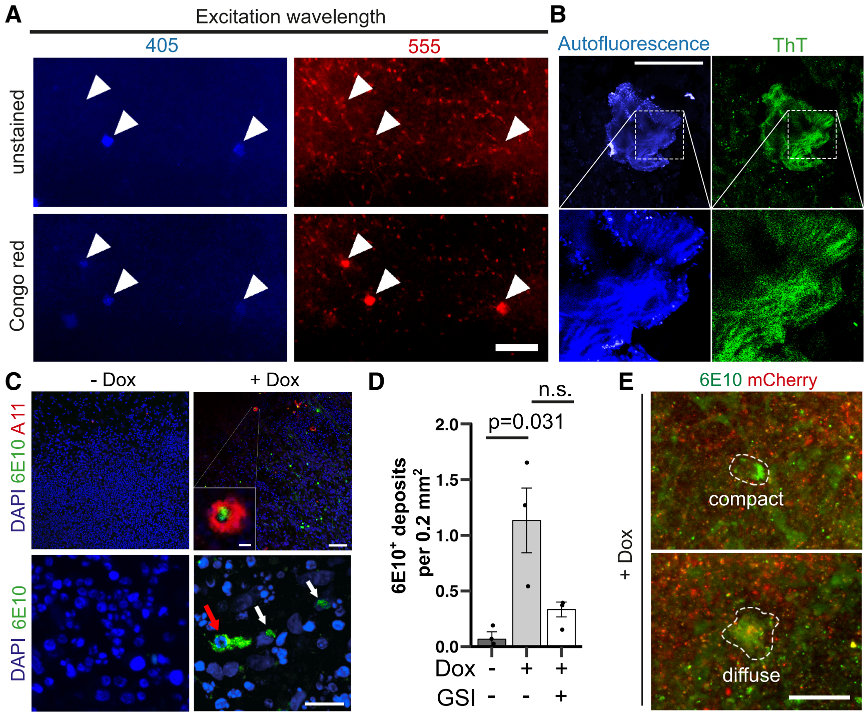

Figure 2. Morphological features of extracellular amyloid deposits

(A) Sequential imaging and staining of a 6-week Dox-induced 3D culture. Fluorescence elicited by 405 nm illumination presented in blue, 488 nm in green, and 555 nm in red in three aggregates (arrowheads). Unstained: imaging after fixation. Congo red: imaging after staining with Congo red. All images from high-density areas. Scale bars, 100 μm.

(B) Confocal image of an aggregate in a 6-week Dox-treated 3D culture. Autofluorescence elicited by 405 nm (emission in blue) and 488 nm (emission in green) in a live culture and after PFA fixation and staining with ThT. Scale bars, 50 μm.

(C) (Upper row) Confocal images of 6-week Dox-induced and non-induced 3D cultures stained with antibodies to Aβ (6E10, green) and Aβ oligomers (A11, red), counterstain DAPI. Scale bars, 200 μm, zoom-in: 10 μm. (Lower row) Confocal images of 6-week Dox-induced and non-induced 3D cultures stained with antibodies to Aβ (6E10, green), counterstain DAPI. Scale bars, 25 μm. Red arrow indicates intracellular deposit. White arrows indicate extracellular deposits.

(D) Quantification of extracellular deposits from (C) normalized to culture surface area. $N = 3$. Data presented as mean ± SEM. Kruskal-Wallis test with Nemenyi's post hoc test.

(E) Anti-Aβ immunofluorescence (6E10, green) and mCherry reporter (red) images of different Aβ deposit types. Scale bars, 100 μm. See also Figure S4.

baseline conditions, with a minimal increase upon Dox treatment, implying Aβ accumulation inside the 3D culture (Figure 1D, right column). However, immunoassay measurements of 2D culture conditioned media after 6 weeks of treatment demonstrated a 7-fold increase in $A\beta_{40}$ (Figure 1E) and a 35-fold increase in $A\beta_{42}$ (Figure 1F). The $A\beta_{42/40}$ ratio—a parameter closely associated with Aβ toxicity—was increased 5-fold to 0.7 (Figure 1G). To test whether Aβ was accumulating inside the 3D cultures, we also analyzed lysates of whole 3D cultures containing only cells and matrix. Despite pronounced variability between our measurements, we found largely elevated concentrations of $A\beta_{40}$ (Figure 1H) and $A\beta_{42}$ (Figure 1I), with an $A\beta_{42/40}$ ratio increase to 1 (Figure 1J). To assess whether Aβ species aggregate within the matrix, solubility fractionation of 4-month-induced cultures was performed. Western blot analysis of the TBS-insoluble fraction revealed prominent total Aβ bands in induced cultures, whereas hardly any Aβ signal was present in uninduced cultures (Figure 1K). Remarkably, Aβ in TBS-insoluble fractions increased 5-fold upon induction (Figures 1K and 1L). Taken together, these data indicate that Aβ species accumulate inside the matrix where they undergo aggregation.

Aβ aggregates show plaque-like properties

Since amyloid deposits are known to exhibit autofluorescence, we initially assessed 405 nm-induced autofluorescence longitudinally in live 3D cultures with and without Dox induction. Starting from 7 to 14 days, Dox-treated cultures developed autofluorescent deposits that intensified over time (Figure S4A). We confirmed the presence of Aβ in the deposits by staining with the 6E10 antibody. 6E10 immunoreactivity was exclusive to the autofluorescent deposits and did not overlap with mCherry signal from the cells, indicating extracellular accumulation (Figure S4B).

To explore neuropathology in this model, we differentiated the neuronal cultures for a total of 8 weeks with Dox treatment for the latter 6 weeks. This is sufficient for functional maturation of lt-NES cell-derived neurons.[21,28] Probing whether retained Aβ had formed plaques in the gel matrix, we fixed 6-week-induced 3D cultures and then stained with Congo red.[29] Since gel cultures developed some inhomogeneities when cultivated for more than 4 weeks, we quantified areas that were structurally intact. Even without staining, aggregate-like structures of ∼20 μm diameter were visible from UV fluorescence in the induced cultures (Figure 2A). After staining with Congo red, aggregate structures became brightly fluorescent in the red channel, while blue fluorescence was diminished (Figure 2A). Similarly, specific binding could be demonstrated with methoxy-X04 (MX04), a dye that detects fibrillar β-sheet aggregates (Figure S4C). Autofluorescent deposits could also be specifically stained with the classical amyloid fibril dye Thioflavin T (ThT; Figures 2B and S4D). Upon closer examination, ThT-stained large deposits (>20 μm) appeared to consist of fibrous bundles (Figure 2B). Uninduced cultures showed no obvious autofluorescent deposits. To probe the presence of oligomeric Aβ inside induced cultures, we performed a co-staining of the oligomer-specific antibody A11 with the APP antibody 6E10. Double-positive structures were found exclusively in induced cultures. Notably, A11 immunoreactivity was often detected as

CellPress
OPEN ACCESS

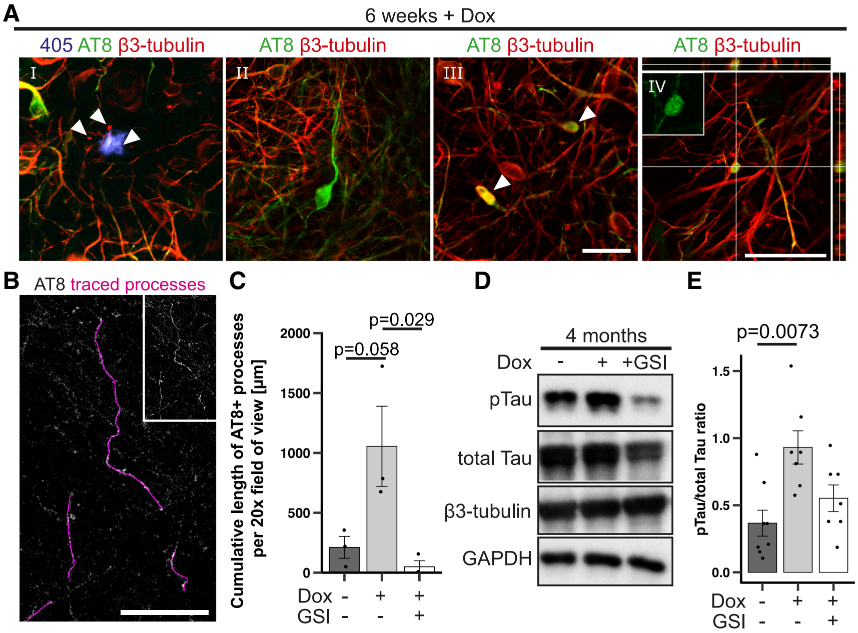

Figure 3. Intracellular accumulation and aggregation of hyperphosphorylated tau

(A) Representative images of various typical stages of neuronal dystrophy in 6-week Dox-induced 3D cultures depicting phospho-tau (AT8, green) and mCherry (red). From left to right: (I) Confocal image of an autofluorescent aggregate surrounded by β3-tubulin-stained neurites (arrowheads). (II) Neuronal "remnant". (III) p-Tau positive bloated neurites (arrowheads). (IV) Axonal swellings (z stack). All scale bars, 50 µm.

(B) Representative image depicting tracing (violet overlay) of strongly p-tau positive (AT8, grayscale) processes for length measurements in 6-week-old, induced 3D cultures. Process length was quantified using the NeuronJ FIJI plugin. Scale bars, 100 µm. Insert shows AT8-positive neurites without tracing overlay.

(C) Quantification of the cumulative distance covered by strongly p-tau positive cellular processes per 20x field of view (n = 3) with three images per experiment and condition. One-way ANOVA with Tukey's post hoc test.

(D) Western blot analysis of 4-month Dox-treated cultures for p-tau (AT8) and total tau.

(E) Corresponding quantification of the p-tau/tau ratio (n = 6–7). Data presented as mean ± SEM. Kruskal-Wallis test with Nemenyi's post hoc test. See also Figure S5.

ring-shaped assemblies around a 6E10-positive core (Figure 2C, upper row).

Small 6E10-positive deposits occurred only upon Dox treatment both inside and outside of cells (Figure 2C, lower row; Figure S4E). Quantification of these deposits revealed a 12-fold difference between uninduced and induced 3D cultures (Figure 2D). Aβ generation from APP depends on APP cleavage by β- and γ-secretase. The small molecule DAPT is a well-characterized GSI and prevents Aβ generation.[30] In accordance with γ-secretase-dependent Aβ generation, GSI application mostly prevented the deposits (Figure 2D). Exploring deposit morphology, we observed 6E10 staining in both small compact and large diffuse structures (Figure 2E). Aβ deposition was confirmed using a second Aβ-specific antibody (D54D2) in conjunction with the pentameric thiophene dye Amytracker 630 (Figure S4F). Collectively, these data show that triple mutant overexpression yields Aβ deposits within the matrix, which share staining and fluorescence properties typically observed in AD plaques.

γ-Secretase inhibition ameliorates elevated tau phosphorylation

Classic AD hallmarks are plaque-like amyloid deposits and intracellular, hyperphosphorylated tau aggregates known as neurofibrillary tangles. To explore the presence of hyperphosphorylated tau protein, we used the phospho-tau antibody AT8. Immunofluorescence analysis in 6-week-induced neurons revealed numerous stained neuronal processes, which were frequently dysmorphic (Figure 3A). UV-bright, likely amyloid, deposits were closely surrounded by neurites, some of which were AT8-positive (Figure 3A^{I}). AT8 staining was also frequently found accumulated in neuronal "ghosts" (neuronal remnants strongly positive for p-tau but negative for other neuronal markers such as β3-tubulin; Figure 3A^{II}), in bulbous neuritic swellings positive for β3-tubulin (Figure 3A^{III}), and in axonal swellings (Figure 3A^{IV}). p-Tau staining in neuronal processes was not restricted to induced cultures but also visible in non-induced conditions. However, whereas uninduced cultures displayed an average cumulative length of 230 µm of strongly p-tau positive processes per 20x field, this value increased to about 1,000 µm in induced cultures. Remarkably, GSI treatment reduced this value to about 50 µm (Figures 3B, 3C, and S5A). The presence of p-tau positive processes was confirmed using a second p-tau-specific monoclonal antibody (Thr181; AT270), which detects paired helical filament-tau and neurofibrillary tangles[31] (Figure S5B). Extending Dox-mediated transgene expression up to 4 months made the difference in AT8 immunoreactivity between induced and uninduced cultures even more apparent (Figure S5C). Western blot analyses of 4-month-old cultures confirmed a significant increase in Tau phosphorylation in induced cultures with the pTau/Tau ratio rising from 0.3 to 0.9, while cultures treated with a GSI exhibited a decreased 0.6 ratio (Figures 3D and 3E). Single dysmorphic neurons in these 4-month-induced cultures displayed typical signs of p-tau pathology, such as asymmetric displacement of the nucleus with a flame-shaped soma, ramification of a strongly p-tau-positive soma, or perinuclear p-tau accumulation, whereas no such features were observed in uninduced or GSI-treated cultures (Figure S5D).

Aggregation of hyperphosphorylated tau is accompanied by conformational changes. During the formation of tau fibrils, tau proteins adopt a "paperclip" conformation that can be recognized as a discontinuous epitope by specific antibodies (such as MC-1).[32] Indeed, in induced cultures, autofluorescent aggregate structures were frequently surrounded by MC-1-positive

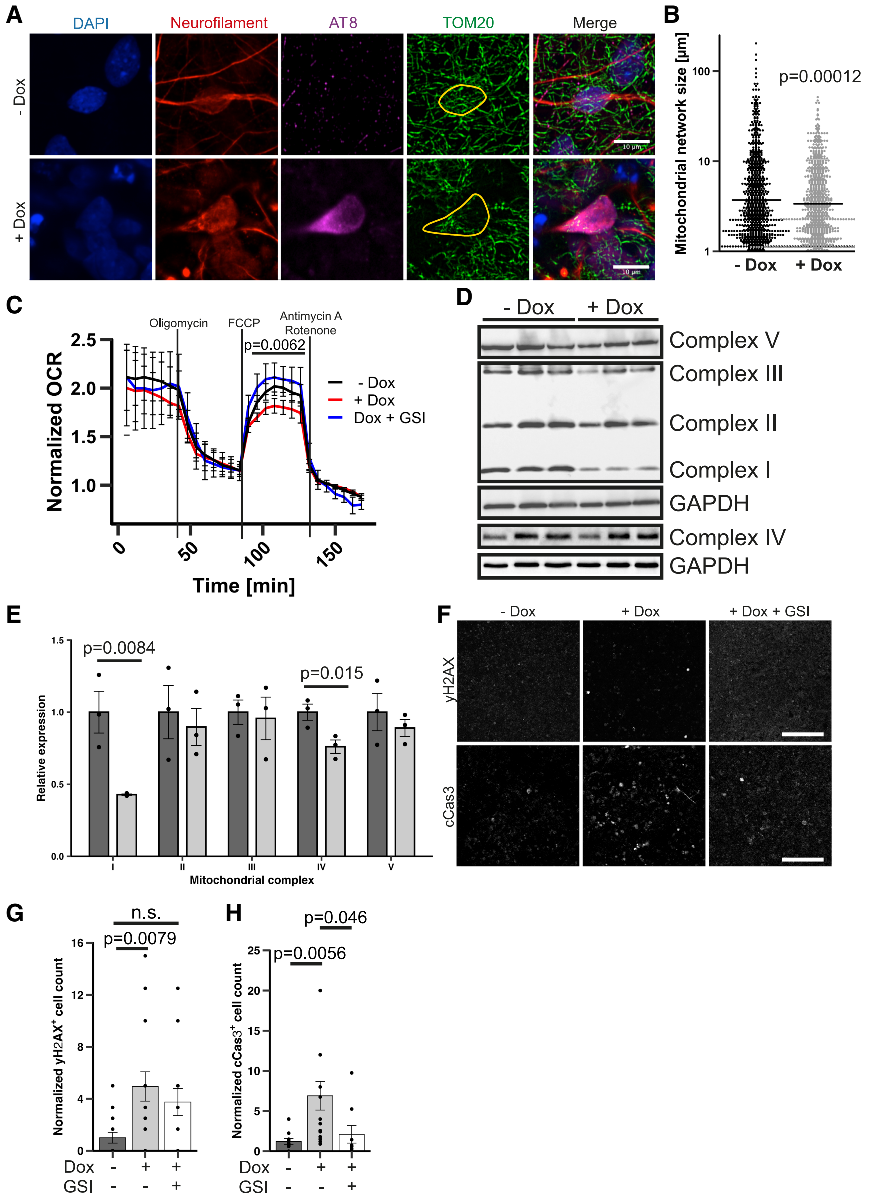

Figure 4. Mitochondrial impairment, DNA damage, and increased apoptosis

(A) Maximum-z projections of representative cells used for mitochondrial quantification. Detection of nuclei (DAPI, blue), neuronal perikarya (neurofilament, red), p-tau (AT8, violet), and mitochondria (TOM20, green). p-Tau positive neuronal perikarya from induced cultures were compared to neurons from uninduced cultures. Neurofilament stain was used to calculate a 3D mask from a z stack representing a single neuronal soma (yellow). Mitochondria inside the mask were quantified 3D tubular reconstruction of each mitochondrial network and determination of the longest non-branching and non-crossing path across the network (mitochondrial network size). Scale bars, 10 μm.

(B) Mitochondrial network size distribution. Cross bars indicate mean. Kruskal-Wallis test with Nemenyi's post hoc test ($n = 14$ cells per treatment condition).

(C) Oxygen consumption rate of 6-week-old 3D cultures in basal conditions (6–42 min), 2 μM oligomycin (48–84 min), 2 μM FCCP (90–126 min), and 1 μM antimycin and rotenone (132–168 min). OCR values were normalized to the mean of the antimycin + rotenone condition. Data presented as mean ± SEM; $n = 3$, 5–6 technical repeats each. Kruskal-Wallis test with Nemenyi's post hoc test.

(D) Western blot analysis of mitochondrial complex proteins from 6-week Dox-treated 3D cultures. Lanes represent biological replicates. (E) Densitometric quantification of mitochondrial complex proteins relative to GAPDH. Quantities were normalized to the mean of the uninduced condition. Data presented as mean ± SEM; $n = 3$; Student's t test.

(F) yH2AX (top, scale bars, 50 μm) and cleaved caspase-3 (bottom, scale bars, 100 μm) staining of 6-week-induced 3D cultures. Single-plane confocal images.

(G) Quantification of yH2AX-positive nuclei (mean ± SEM; $n = 3$; 4–5 images per condition; Kruskal-Wallis with Nemenyi's post hoc test).

(H) Quantification of cCas3-positive cells (mean ± SEM; $n = 3$–4; ANOVA with Tukey's post hoc test).

See also Figure S5.

neurites ending in blotch-like terminals on, or inside of the aggregate. The dystrophic terminals were strongly labeled by Thioflavin T (Figure S5E). Additionally, single highly MC-1-reactive neurons were found. In GSI-treated cultures, neither conglomerates with autofluorescence nor MC-1 reactivity could be detected (Figure S5F).

Taken together, these observations indicate that overexpression of the $APP_{Swe/Lon\text{-}PSEN1\Delta E9}$ triple mutant induces both, Aβ and tau pathology within our lt-NES cell-based 3D matrix system.

Aberrant mitochondrial morphology and respiration are associated with neurodegeneration in AD cultures

Altered mitochondrial dynamics have been repeatedly implicated in AD.[33] Mitochondrial connectivity was evaluated morphologically and based on the shape and length of uninterrupted path without branching or crossing within other mitochondrial units. To assess the mitochondrial network, we employed 6-week Dox-treated and control 3D cultures. Using TOM20 staining, we compared the mitochondrial network size in p-tau positive neuronal cell bodies of Dox-treated cultures with their non-Dox-treated counterparts (Figure 4A). AT8-positive neurons from induced cultures showed clear abnormal mitochondrial morphology compared to neurons from uninduced cultures. The average mitochondrial network size per neuronal soma was reduced by approximately 30% in AT8-positive neurons (Figure 4B). To determine whether altered mitochondrial morphology was associated with respiratory changes, we measured oxygen consumption rate (OCR) in 6-week induced

and control cultures. All tested cultures displayed a typical profile during Seahorse analysis after normalization to the antimycin A + rotenone condition. However, induced cultures had significantly lower respiratory capacity than controls (Figure 4C). We also observed a lower OCR in induced cultures in the non-normalized dataset, which could reflect lower cell numbers or less healthy cells (Figure S5G). Since Dox has been reported to cause mitochondrial impairment in various cell lines, we assayed short-term toxicity of the induction paradigm by applying a wide range of doxycycline concentrations (0.01–20 μg/mL) to control lt-NES neurons for 7 days, which did not result in detrimental changes to the OCR (Figure S5H).[34] Furthermore, applying GSI during Dox-induction prevented the reduced OCR phenotype despite the presence of Dox (Figure 4C). Looking for a mechanistic cause underlying the decreased respiratory capacity, we performed western blot analysis of 6-week Dox-treated and control 3D cultures and found a significant reduction in subunits of the respiratory chain complexes I and IV (60% and 25%, respectively). In contrast, complexes II, III, and V were unaltered (Figures 4D and 4E).

Since amyloid, p-tau, and mitochondrial defects are signatures of AD, 6-week-treated 3D cultures were further scrutinized for neurodegeneration. Immunostaining for phosphorylated histone 2A (γH2AX), which indicates DNA double-strand breaks, revealed a 5-fold increase in the number of labeled cells in induced vs. uninduced cultures. Concomitant application of Dox and a GSI reduced the DNA damage phenotype below the level of statistical significance, but the trend toward an elevation remained (Figure 4F, upper row, 4G). We subsequently performed an immunofluorescence staining with an antibody to the apoptosis marker cleaved (activated) caspase 3 (cCas3). Quantification revealed a significant 5-fold increase of cCas3-positive cells over uninduced cultures without major changes of nuclear morphology (Figure S5I). GSI-treated induced cultures displayed significantly lower numbers of cCas3-positive cells with no significant difference to uninduced cultures (Figures 4F, lower row, and 4H).

These data show that overexpression of the $APP_{Swe/Lon\text{-}PSEN1\Delta E9}$ mutant in our 3D system results in mitochondrial dysfunction and neurodegeneration reflected by DNA damage and increased apoptosis.

Amenability of 3D cultures to microglia integration

Microglia are known to play a pivotal role in neurodegenerative diseases such as AD.[35–37] We sought to test whether our 3D culture system was suitable for studying the response of microglia to AD-related pathology. As an initial step in this direction, we generated iPSC-derived microglia (iPSdMiG) according to our previously published protocol[38] and added them to 6-week-old 3D cultures, which were then maintained for another 5 days.[38] To first assess how iPSdMiG would respond to the 3D culture environment and exposure to Dox, cells were added to cell-free 3D cultures and maintained in the presence or absence of Dox. The iPSdMiG readily entered the 3D matrix and spread across the culture volume. Microglial marker expression remained similar between 2D and 3D conditions, and also upon exposure to Dox in 3D culture (Figure S6A). Staining for cCAS3 revealed a very low percentage of apoptotic cells with no significant differences between Dox-free and Dox-treated cultures (0.49 ± 0.21% vs. 1.04 ± 0.48%, respectively; Figures S6B and S6C). These data indicate that neither culturing in the 3D matrix nor treatment with Dox leads to an overt induction of apoptosis in iPSdMiG.

Microglia are specialized phagocytes of the CNS with a number of homeostatic function including clearance of dying cells[39] and pathologically aggregated proteins.[40] To assess these properties, we started 3D co-cultures of 6-week-doxycycline-induced $APP_{Swe/Lon\text{-}PSEN1\Delta E9}$ triple mutant lt-NES cell-derived neurons with iPSdMiG. Five days after co-culture, IBA1+ iPSdMiG had taken up cCAS3 immunoreactive material in both Dox-induced and non-Dox-induced cultures, where it comprised up to 10% of the cross-sectional areas of the IBA1+ cells (Figures 5A and 5D). Double labeling for IBA1 and 6E10 revealed that iPSdMiG had also phagocytosed Aβ, the level of which was increased 3-fold in Dox-induced 3D cultures. We quantified this process by measuring the area of 6E10-positive specks colocalizing with IBA-1-positive cells (Figures 5B and 5E). Intracellular location of Aβ material was confirmed by confocal imaging using z stack reconstruction (Figure 5C).

We then wondered whether co-culture with microglia would affect the overall Aβ42/40 ratio in the 3D cultures and their supernatant. After 5 days of co-culture, we observed a trend toward decreased Aβ42/40 ratios in cell+matrix lysates of both uninduced and induced co-cultures, which was more evident in induced cultures (Figure 5F). In the supernatant of Dox-induced cultures, this effect became stronger and revealed a significant reduction of the Aβ42/40 ratio in the presence of iPSdMiGs (Figure 5G). Notably, co-cultivation with Dox-treated neuronal cultures did not induce secretion of the proinflammatory cytokines TNFα and IL1β, suggesting a predominantly non-inflammatory uptake at this time point (Figures S6D and S6E).

Taken together, these data indicate that our 3D culture system is amenable to the integration of iPSC-derived microglia, which phagocytose Aβ and decrease Aβ42/40 ratios.

DISCUSSION

Despite extensive research into AD, current mechanistic insights have proven insufficient for effective therapeutic interventions. One major hurdle is the lack of suitable model systems for basic research and drug development. In light of the recent regulatory approval of the anti-Aβ antibodies lecanemab and donanemab despite limited efficacy and substantial side effects, as well as the removal of aducanumab from the market, mouse models have delivered low predictive value for human treatments.[41] Attempts to model AD in 2D iPSC-derived cultures were not able to recapitulate plaque-like amyloid deposition and p-tau pathology in a comprehensive fashion, although studies in trisomy 21 iPS cell neurons found small-scale amyloid deposition when focusing on clumps of neural cell bodies.[42] 3D culture paradigms are increasingly employed to circumvent these limitations by mimicking a more *in vivo*-like environment. Most notably, cerebral organoids and chimeric transplants generate more mature cell types and can yield subtle disease phenotypes when based on patient iPS cells or individual AD mutations.[5–7] However, these models are complex, expensive and as of yet unsuitable

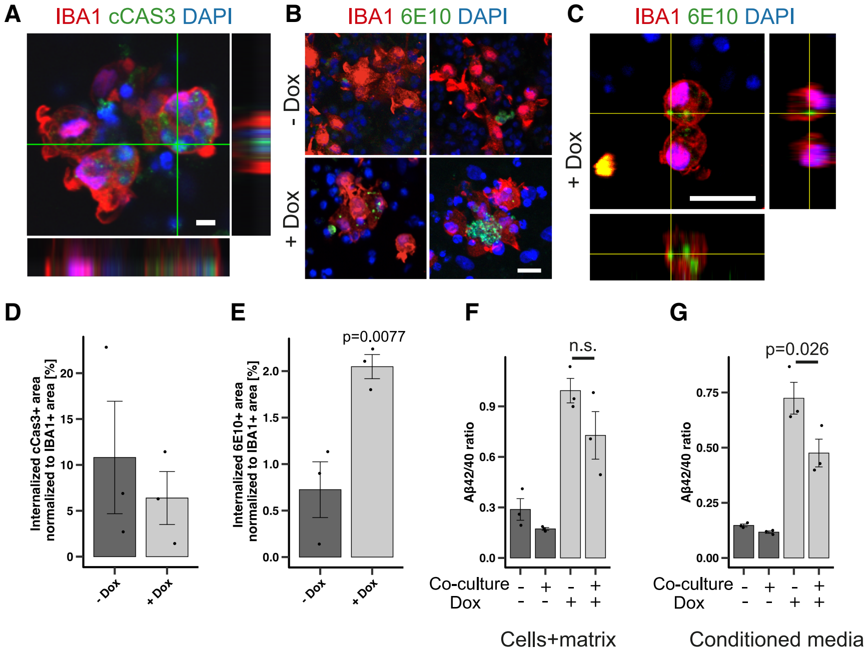

Figure 5. Microglial co-culture and Aβ uptake

(A and D) Representative confocal images and quantification of cCAS-positive material internalized by iPSdMiG in 3D co-cultures with lt-NES-derived neurons, normalized to total IBA1-positive area ($n = 3$). Scale bars, 20 μm.

(B and E) Representative confocal images and quantification of 6E10-positive material internalized by iPSdMiG in 3D co-cultures with lt-NES-derived neurons, normalized to total IBA1-positive area ($n = 3$; Student's t test, one-tailed, unpaired). Scale bars, 20 μm.

(C) Representative 3D reconstruction of a confocal z stack showing internalized 6E10-positive material within the cytoplasm of IBA1-positive iPSdMiG. Scale bars, 25 μm.

(F and G) Ratio of Aβ42/40 determined within cell-containing matrix pellet (left) and supernatant of 3D neuronal mono-cultures (right) compared to co-cultures with iPSdMiG in both Dox-induced and non-Dox-induced conditions ($n = 3$; ANOVA with Tukey's post hoc test). All data presented as mean ± SEM. See also Figure S6.

for classical compound screens. Alternative 3D cultures have been used to mimic a 3D tissue environment, thereby enabling acquisition of more physiological 3D cell morphologies and 3D cell-cell interactions.[43] Moreover, flexible scaffolds used for such cultures can be functionalized and, e.g., supplemented with neurogenesis-relevant extracellular constituents as needed.[44–46]

For this study, human iPS cells were genetically engineered to conditionally overexpress $APP_{Swe/Lon}$ and *PSEN1(ΔE9)* ($APP_{Swe/Lon\text{-}PSEN1\Delta E9}$) from a genomic safe-harbor locus to enable highly standardized and controllable Aβ generation. Such an inducible system prevents developmental side effects from constitutive overexpression of FAD mutations,[17,18] including, e.g., an early impact on Notch signaling by altered γ-secretase activity.[47] Previous studies of some of us have employed RenVM-based 3D cultures for assessing AD-related pathologies.[13,23] However, the immortalized nature of these oncogene-expressing cells limits their authenticity.[16] In AD, neurons appear to be mostly responsible for plaque generation and p-tau pathology, although an extensive body of literature also addresses contributions from other cell types including astrocytes, microglia, or macrophages.[48–50] Our 3D cultures contain both neurons (β3-tubulin, GABA, and vGlut1) and astrocytes (S100β) at very high densities, thereby providing a suitable composition for AD disease modeling *in vitro*.

The fidelity of our inducible system was confirmed in 2D cultures, where Dox induction increased $A\beta_{40}$ levels in the supernatant 5-fold and $A\beta_{42}$ levels 40-fold with an overall 8-fold increase of the $A\beta_{42/40}$ ratio, changes that are clinically associated with drastically increased pathogenicity.[51] The increase in $A\beta_{40}$ generation was 2-fold less than previously reported using the same cassette, but the $A\beta_{42}$ change surpassed earlier results approximately 4-fold.[13] In a more recent follow-up, members of the Kim lab compared clonal cell lines with distinct and consistent $A\beta_{42/40}$ ratios from 0.05 to 1.4, and found a strong correlation to p-tau pathology starting from a ratio of approx. 0.3.[14] Thus, the $APP_{Swe/Lon\text{-}PSEN1\Delta E9}$ system generates an $A\beta_{42/40}$ ratio well inside the established pathogenic range *in vitro*. Nonetheless, our model is artificial in its overexpression of mutant *APP*. This stands in stark contrast to the human brain, where most familial Alzheimer's mutations actually reduce the total amount of Aβ being generated (while the Aβ42/40 ratio increases).

Since Alzheimer's pathology primarily impacts adult neurons, we differentiated our 3D cultures for a minimum of 8 weeks. At this time point, lt-NES neurons have been shown to be electrically active and form functional synapses.[21,28] While these cells offer great advantages with respect to expandability and robust neuronal and glial differentiation across many passages, they, together with a number of other growth factor-expanded cell populations, exhibit posteriorization with a regional identity around the mid-hind brain boundary, not cortical neurons. Targeting cortical neuron populations is an opportunity for further improvement in future work. Upon induction of triple mutant expression, we first observed accumulating autofluorescent deposits that stained positive for Aβ, and then strong accumulation of TBS- and SDS-insoluble Aβ in the matrix compartment over time, whereas 2D cultures secreted Aβ into the supernatant. By week 8, induced 3D cultures occasionally contained large autofluorescent deposits (20–100 μm). Staining with amyloid dyes (ThT, Congo red, MX04, Amytracker 630), Aβ antibodies (6E10 and D54D2), oligomer-specific antibodies (A11), and morphological analysis (fibrous interior, "burned out") confirmed their plaque-like nature.[52,53] Imaging of UV-induced autofluorescence together with Thioflavin T staining unveiled a near-perfect overlap in fibrillar areas in the core of the aggregate, whereas no ThT staining was found in globular or amorphous peripheral

regions.[54] Amyloid aggregates were sometimes associated with other pathology as shown by rings of Aβ oligomers surrounding a 6E10-positive core, and by dystrophic neurites surrounding autofluorescent plaque-like structures.

Induced cultures also contained neurons with somatodendritic p-tau accumulation (AT8, AT270). High-resolution imaging uncovered dysmorphic, strongly p-tau positive stretches of processes as well as neurons with highly characteristic flame-shaped cell bodies, matching histopathological data from AD patient brains.[55] Tracing of p-tau positive processes revealed a 5-fold increased segment length in induced over uninduced cultures. Additionally, treatment with a GSI prevented p-tau reactivity almost completely.[56] Further features of neuronal dystrophy in our model include p-tau "husks", dystrophic neurites, dystrophic terminals and axonal swellings similar to previous reports of *in vitro* tauopathy.[56] After four months of treatment, the p-tau/tau ratio had increased from 0.3 to 0.9. Like in AD brain tissue, singular strongly MC-1-positive whole cells appeared surrounded by negative cells.[55] Finally, induced cultures contained many "beads-on-a-string" formations of strongly MC-1-reactive presumptive neurite debris. Taken together, these results establish that both amyloid and p-tau pathologies in our model can be elicited by only manipulating Aβ generation. The presented model supports the amyloid cascade hypothesis as both major AD hallmarks occur sequentially and progressively and that inhibiting APP processing can effectively prevent both phenotypes.[2] However, the general absence of "adult" tau splice isoforms in iPS-derived models is a major limitation on the severity of p-tau pathology. Recently, iPS-based spheroid models approached 20% 4R tau (compared to 50% in adult brain), but only after 6 months of differentiation.[57,58] Thus, future improvements in differentiation paradigms might enable robust recapitulation of NFTs.

Beyond histopathological hallmarks, AD is characterized by metabolic and functional impairment of affected cells. For instance, mitochondria in AD display increased fragmentation, reduced respiratory chain proteins and reduced respiratory capacity.[59–61] Interestingly, mitochondrial network changes were only detectable in cells with somatodendritic p-tau accumulation as has been found in AD patient brains.[62] Such a selective mitochondrial pathology also serves as an internal control for potential side effects of the Tet-On system since Dox has been reported to affect mitochondrial translation at very high concentrations.[34] To assess mitochondrial functionality, we carried out Seahorse OCR measurements in matrix 3D cultures. We detected a clear reduction in maximum respiration rate in induced cultures, which may reflect lower mitochondrial bioenergetic plasticity and increased susceptibility to metabolic stress. We also observed a subtle reduction in the expression of complex I and IV subunits in induced cultures, which could explain the reduction in maximum respiratory capacity. Importantly, these effects were fully rescued by blocking APP processing with two different GSIs, further supporting a role for the amyloid cascade rather than potential Dox toxicity.

The final stage of AD features extensive neuronal loss and cognitive decline. Up to this point, Aβ-based mouse models have mostly failed to recapitulate widespread neurodegeneration, while studies involving particular humanized tau isoforms showed pronounced neurotoxicity.[63,64] *In vitro* models, on the other hand, typically comprise artificial cell populations highly resistant to cell death due to oncogene expression or insufficiently strong pathology for late-stage disease modeling.[13,65] On the other extreme, a comparable 3D approach showed neurodegeneration in late-onset AD neurons derived from aged fibroblasts by direct reprogramming.[66] However, the time window for analysis in this model is only about 10 days as the observed degeneration is rapid. In the presented model, induction for 6 weeks increased markers of apoptosis and severe DNA damage 5-fold. It should be noted here that GSI treatment prevented excess apoptosis as shown by cleaved caspase-3 but did not fully ameliorate DNA damage indicated by γH2AX. A possible explanation for this effect is that GSI treatment interrupts Notch signaling, which is involved in the regulation of DNA repair, and reduces the fraction of astrocytes in the cultures, which contributes to neuronal stress.[24,67] The presence of apoptotic cells suggests ongoing degenerative processes, which qualify the model for testing of neurotoxicity-reducing treatments in long-term paradigms. The exact mechanisms underlying the road to cell death in our model have not yet been elucidated and might provide further insight into AD pathogenesis. For example, the system enables studies of plaque development with repeated imaging sessions over weeks in the presence of fibril dyes or antibodies, similar to what could only be achieved in mice with "cortical windows." During that process, early-stage pathologies could be investigated using, e.g., scRNAseq and further metabolic characterization to better understand disease initiation.

Inclusion of microglial cells in the model builds upon the slow degenerative phenotype. IPSdMiG cells in Geltrex with and without Dox show robust survival in 3D cultures with an approximate 1% cCAS3-positive rate. Within 5 days of entering the gel cultures, iPSdMiG phagocytose apoptotic cells and accumulate Aβ (6E10), thus demonstrating typical functionality. Notably, increased phagocytosis in the induced condition was specific to Aβ, whereas there was no significant difference in the uptake of dead cells. This suggests that dead cells and Aβ are distinct triggers for microglial phagocytosis. Furthermore, Aβ measurements in the culture supernatant, but not in the pellet (matrix + cells), showed a significant reduction in the $A\beta_{42/40}$ ratio in induced cultures in the presence of iPSdMiG, hinting at clearance of Aβ by microglial cells. This reduction was not observed in the pellets of neuronal monocultures compared to co-cultures under Dox treatment. Likely, the pellet Aβ includes engulfed but undigested material found within the microglial cells. It should be noted that the observed changes upon iPSdMiG addition were rapid. Longer co-cultivation might present different outcomes. Ideally, iPSdMiG should be matured together with the neuronal and astrocytic components to better approximate the brain.

From a broader perspective, our model could be employed to study the relationship between neurite degeneration and nearby plaque formation, as well as the formation and spread of emerging tau tangles. Upon inclusion of appropriate markers, seeding experiments could be implemented easily by mixing samples into the gel matrix before solidification. Lastly, both multi-electrode array (MEA) technology as well as dye and protein voltage sensors might be employed to elucidate the functional impairments of mounting AD pathology on the neuronal

circuits in the culture. This method has been used previously to study early Aβ42-driven synaptotoxicity and is likely to yield new insights in the later disease stages modeled here.[68] Based on these perspectives, we are confident that the model will prove useful to explore mid-to-late-stage AD mechanisms and therapeutic approaches in an authentic, standardized, and modular human setting.

Recent advances in amyloid-targeting drugs as well as their unexpected side effects and limited efficacy in symptomatic AD underscore the importance of this type of late-stage model.

Limitations of the study

While our model system is based on authentic neurons derived from iPSCs, it relies on the overexpression of three pathogenic *APP* and *PSEN1* variants; a condition that is not observed in AD patients. Furthermore, APP overexpression results in a strong increase of Aβ levels, whereas AD patients often exhibit a decreased total amount of Aβ, but with pathologically altered isoform ratios. Since epigenetic aging signatures are removed during reprogramming, iPSC models in general are limited with respect to assessing the impact of aging on the onset or progression of the *in vitro* pathology. General limitations of such *in vitro* models include the lack of the broad cell type heterogeneity, extracellular matrix composition, and histoarchitecture of the brain as well as absence of systemic modulation, e.g., via the vascular, hematopoietic, and peripheral immune systems.

RESOURCE AVAILABILITY

Lead contact

Requests for further information and resources should be directed to and will be fulfilled by the lead contact, Oliver Brüstle (brustle@uni-bonn.de).

Materials availability

All unique/stable reagents generated in this study are available from the lead contact with a completed materials transfer agreement.

Data and code availability

- All data reported in this paper will be shared by the lead contact upon request.
- This paper does not report original codes.
- Any additional information required to reanalyze the data reported in this paper is available from the lead contact upon request.

ACKNOWLEDGMENTS

We thank Dr. Su-Chun Zhang (Center for Neurologic Diseases, Sanford Burnham Prebys, La Jolla, CA 92037, USA) for kindly providing the AAVS targeting vector and corresponding TALENs and Peter Davies for providing the MC-1 antibody. Furthermore, we thank Cornelia Thiele, Melanie Bloschies, and Tamara Bechler for outstanding technical support, and Karlheinz Baumann and Fiona Grüninger for contributions to data analysis. This work was supported by the German Federal Ministry of Education and Research (grant 01EK1603A-Neuro2D3), F. Hoffmann-La Roche Ltd., and the ADAPTED consortium, which has received funding from the Innovative Medicines Initiative 2 Joint Undertaking under grant agreement no. 115975. This Joint Undertaking received support from the European Union's Horizon 2020 research and innovation program and the European Federation of Pharmaceutical Industries and Associations. D.Y.K. was supported by the Cure Alzheimer's Fund (2023A069137). The contribution of V.K. was supported by the EKFS-scholarship Q-611.2954 (BonnIE Program) of the University of Bonn Medical Faculty. O.B. is a member of the Cluster of Excellence ImmunoSensation2—EXC 2151-390873048.

AUTHOR CONTRIBUTIONS

M.H. and V.K., collection and assembly of data, data analysis and interpretation, and manuscript writing; G.C., A.P., F.B., B.W., and K.W., collection and assembly of data, data analysis and interpretation; S.K., data analysis and interpretation; D.K. and D.B., data interpretation and manuscript writing; M.P. and O.B., conception and design, data interpretation, and manuscript writing.

DECLARATION OF INTERESTS

O.B. is a co-founder and shareholder of LIFE & BRAIN GmbH.

STAR★METHODS

Detailed methods are provided in the online version of this paper and include the following:

- KEY RESOURCES TABLE
- EXPERIMENTAL MODEL AND STUDY PARTICIPANT DETAILS
  - IPSC culture and quality control
- METHOD DETAILS
  - Karyotyping
  - Generation of triple-mutant iPSCs
  - Generation of 3D cultures
  - Immunofluorescence analyses
  - Imaging of amyloid and p-tau deposition
  - Quantification of Aβ species
  - Western blotting
  - Mitochondrial analysis
  - Microglia co-culture
- QUANTIFICATION AND STATISTICAL ANALYSIS
  - Statistical analysis

SUPPLEMENTAL INFORMATION

Supplemental information can be found online at https://doi.org/10.1016/j.crmeth.2026.101365.

Received: April 29, 2025
Revised: October 29, 2025
Accepted: February 19, 2026
Published: March 30, 2026

REFERENCES

1. Tanzi, R.E., and Bertram, L. (2005). Twenty years of the Alzheimer's disease amyloid hypothesis: a genetic perspective. Cell *120*, 545–555. https://doi.org/10.1016/j.cell.2005.02.008.
2. Selkoe, D.J., Hardy, J., Selkoe, D., and Hardy, J. (2016). The amyloid hypothesis of Alzheimer's disease at 25 years. EMBO Mol. Med. *8*, 595–608. https://doi.org/10.15252/emmm.201606210.
3. Cenini, G., Hebisch, M., Iefremova, V., Flitsch, L.J., Breitkreuz, Y., Tanzi, R.E., Kim, D.Y., Peitz, M., and Brüstle, O. (2021). Dissecting Alzheimer's disease pathogenesis in human 2D and 3D models. Mol. Cell. Neurosci. *110*, 103568. https://doi.org/10.1016/j.mcn.2020.103568.
4. Lomoio, S., Pandey, R.S., Rouleau, N., Menicacci, B., Kim, W., Cantley, W.L., Haydon, P.G., Bennett, D.A., Young-Pearse, T.L., Carter, G.W., et al. (2023). 3D bioengineered neural tissue generated from patient-derived iPSCs mimics time-dependent phenotypes and transcriptional features of Alzheimer's disease. Mol. Psychiatry *28*, 5390–5401. https://doi.org/10.1038/s41380-023-02147-3.
5. Lin, Y.-T., Seo, J., Gao, F., Feldman, H.M., Wen, H.-L., Penney, J., Cam, H.P., Gjoneska, E., Raja, W.K., Cheng, J., et al. (2018). APOE4 causes widespread molecular and cellular alterations associated with Alzheimer's

disease phenotypes in human iPSC-derived brain cell types. Neuron *98*, 1141–1154.e7. https://doi.org/10.1016/j.neuron.2018.05.008.

6. Najm, R., Zalocusky, K.A., Zilberter, M., Yoon, S.Y., Hao, Y., Koutsodendris, N., Nelson, M., Rao, A., Taubes, A., Jones, E.A., and Huang, Y. (2020). In vivo chimeric Alzheimer's disease modeling of Apolipoprotein E4 toxicity in human neurons. Cell Rep. *32*, 107962. https://doi.org/10.1016/j.celrep.2020.107962.
7. Zhao, J., Fu, Y., Yamazaki, Y., Ren, Y., Davis, M.D., Liu, C.-C., Lu, W., Wang, X., Chen, K., Cherukuri, Y., et al. (2020). APOE4 exacerbates synapse loss and neurodegeneration in Alzheimer's disease patient iPSC-derived cerebral organoids. Nat. Commun. *11*, 5540. https://doi.org/10.1038/s41467-020-19264-0.
8. Mungenast, A.E., Siegert, S., and Tsai, L.-H. (2016). Modeling Alzheimer's disease with human induced pluripotent stem (iPS) cells. Mol. Cell. Neurosci. *73*, 13–31. https://doi.org/10.1016/j.physbeh.2017.03.040.
9. Penney, J., Ralvenius, W.T., and Tsai, L.H. (2020). Modeling Alzheimer's disease with iPSC-derived brain cells. Mol. Psychiatry *25*, 148–167. https://doi.org/10.1038/s41380-019-0468-3.
10. Bubnys, A., and Tsai, L.-H. (2022). Harnessing cerebral organoids for Alzheimer's disease research. Curr. Opin. Neurobiol. *72*, 120–130. https://doi.org/10.1016/j.conb.2021.10.003.
11. Balusu, S., Horré, K., Thrupp, N., Craessaerts, K., Snellinx, A., Serneels, L., T'Syen, D., Chrysidou, I., Arranz, A.M., Sierksma, A., et al. (2023). MEG3 activates necroptosis in human neuron xenografts modeling Alzheimer's disease. Science *381*, 1176–1182. https://doi.org/10.1126/science.abp9556.
12. Fernandes, S., Revanna, J., Pratt, J., Hayes, N., Marchetto, M.C., and Gage, F.H. (2024). Modeling Alzheimer's disease using human cell derived brain organoids and 3D models. Front. Neurosci. *18*, 1434945. https://doi.org/10.3389/fnins.2024.1434945.
13. Choi, S.H., Kim, Y.H., Hebisch, M., Sliwinski, C., Lee, S., D'Avanzo, C., Chen, H., Hooli, B., Asselin, C., Muffat, J., et al. (2014). A three-dimensional human neural cell culture model of Alzheimer's disease. Nature *515*, 274–278. https://doi.org/10.1038/nature13800.
14. Kwak, S.S., Washicosky, K.J., Brand, E., von Maydell, D., Aronson, J., Kim, S., Capen, D.E., Cetinbas, M., Sadreyev, R., Ning, S., et al. (2020). Amyloid-β42/40 ratio drives tau pathology in 3D human neural cell culture models of Alzheimer's disease. Nat. Commun. *11*, 1377. https://doi.org/10.1038/s41467-020-15120-3.
15. Park, J., Wetzel, I., Marriott, I., Dréau, D., D'Avanzo, C., Kim, D.Y., Tanzi, R.E., and Cho, H. (2018). A 3D human triculture system modeling neurodegeneration and neuroinflammation in Alzheimer's disease. Nat. Neurosci. *21*, 941–951. https://doi.org/10.1038/s41593-018-0175-4.
16. McMahon, S.B. (2010). Emerging concepts in the analysis of transcriptional targets of the MYC oncoprotein: Are the targets targetable? Genes Cancer *1*, 560–567. https://doi.org/10.1177/1947601910379011.
17. Zaletel, I., Schwirtlich, M., Perović, M., Jovanović, M., Stevanović, M., Kanazir, S., and Puškaš, N. (2018). Early impairments of hippocampal neurogenesis in 5xFAD mouse model of Alzheimer's disease are associated with altered expression of SOXB transcription factors. J. Alzheimers Dis. *65*, 963–976. https://doi.org/10.3233/JAD-180277.
18. Wu, Y., Zhang, S., Xu, Q., Zou, H., Zhou, W., Cai, F., Li, T., and Song, W. (2016). Regulation of global gene expression and cell proliferation by APP. Sci. Rep. *6*, 22460. https://doi.org/10.1038/srep22460.
19. Qian, K., Huang, C.T.L., Chen, H., Blackbourn, L.W., Chen, Y., Cao, J., Yao, L., Sauvey, C., Du, Z., and Zhang, S.-C. (2014). A simple and efficient system for regulating gene expression in human pluripotent stem cells and derivatives. Stem Cells *32*, 1230–1238. https://doi.org/10.1002/stem.1653.
20. Papapetrou, E.P., and Schambach, A. (2016). Gene insertion into genomic safe harbors for human gene therapy. Mol. Ther. *24*, 678–684. https://doi.org/10.1038/mt.2016.38.
21. Koch, P., Opitz, T., Steinbeck, J. a, Ladewig, J., and Brüstle, O. (2009). A rosette-type, self-renewing human ES cell-derived neural stem cell with potential for in vitro instruction and synaptic integration. Proc. Natl. Acad. Sci. USA *106*, 3225–3230. https://doi.org/10.1073/pnas.0808387106.
22. Falk, A., Koch, P., Kesavan, J., Takashima, Y., Ladewig, J., Alexander, M., Wiskow, O., Tailor, J., Trotter, M., Pollard, S., et al. (2012). Capture of neuroepithelial-like stem cells from pluripotent stem cells provides a versatile system for in vitro production of human neurons. PLoS One *7*, e29597. https://doi.org/10.1371/journal.pone.0029597.
23. Kim, Y.H., Choi, S.H., D'Avanzo, C., Hebisch, M., Sliwinski, C., Bylykbashi, E., Washicosky, K.J., Brüstle, O., Tanzi, R.E., and Kim, D.Y. (2015). A 3D human neural cell culture system for modeling Alzheimer's disease. Nat. Protoc. *10*, 985–1006. https://doi.org/10.1038/nprot.2015.065.A.
24. Borghese, L., Dolezalova, D., Opitz, T., Haupt, S., Leinhaas, A., Steinfarz, B., Koch, P., Edenhofer, F., Hampl, A., and Brüstle, O. (2010). Inhibition of notch signaling in human embryonic stem cell-derived neural stem cells delays G1/S phase transition and accelerates neuronal differentiation in vitro and in vivo. Stem Cells *28*, 955–964. https://doi.org/10.1002/stem.408.
25. Kondo, T., Asai, M., Tsukita, K., Kutoku, Y., Ohsawa, Y., Sunada, Y., Imamura, K., Egawa, N., Yahata, N., Okita, K., et al. (2013). Modeling Alzheimer's disease with iPSCs reveals stress phenotypes associated with intracellular Aβ and differential drug responsiveness. Cell Stem Cell *12*, 487–496. https://doi.org/10.1016/j.stem.2013.01.009.
26. Israel, M. a, Yuan, S.H., Bardy, C., Reyna, S.M., Mu, Y., Herrera, C., Hefferan, M.P., Van Gorp, S., Nazor, K.L., Boscolo, F.S., et al. (2012). Probing sporadic and familial Alzheimer's disease using induced pluripotent stem cells. Nature *482*, 216–220. https://doi.org/10.1038/nature10821.
27. Koch, P., Tamboli, I.Y., Mertens, J., Wunderlich, P., Ladewig, J., Stüber, K., Esselmann, H., Wiltfang, J., Brüstle, O., and Walter, J. (2012). Presenilin-1 L166P mutant human pluripotent stem cell-derived neurons exhibit partial loss of γ-secretase activity in endogenous amyloid-β generation. Am. J. Pathol. *180*, 2404–2416. https://doi.org/10.1016/j.ajpath.2012.02.012.
28. Grønning Hansen, M., Laterza, C., Palma-Tortosa, S., Kvist, G., Monni, E., Tsupykov, O., Tornero, D., Uoshima, N., Soriano, J., Bengzon, J., et al. (2020). Grafted human pluripotent stem cell-derived cortical neurons integrate into adult human cortical neural circuitry. Stem Cells Transl. Med. *9*, 1365–1377. https://doi.org/10.1002/sctm.20-0134.
29. Puchtler, H., Sweat, F., and Levine, M. (1962). On the binding of Congo red by amyloid. J. Histochem. Cytochem. *10*, 355–364. https://doi.org/10.1177/10.3.355.
30. Dovey, H.F., John, V., Anderson, J.P., Chen, L.Z., de Saint Andrieu, P., Fang, L.Y., Freedman, S.B., Folmer, B., Goldbach, E., Holsztynska, E.J., et al. (2001). Functional gamma-secretase inhibitors reduce beta-amyloid peptide levels in brain. J. Neurochem. *76*, 173–181. https://doi.org/10.1046/j.1471-4159.2001.00012.x.
31. van Eersel, J., Ke, Y.D., Liu, X., Delerue, F., Kril, J.J., Götz, J., and Ittner, L.M. (2010). Sodium selenate mitigates tau pathology, neurodegeneration, and functional deficits in Alzheimer's disease models. Proc. Natl. Acad. Sci. USA *107*, 13888–13893. https://doi.org/10.1073/pnas.1009038107.
32. Jicha, G.A., Bowser, R., Kazam, I.G., and Davies, P. (1997). Alz-50 and MC-1, a new monoclonal antibody raised to paired helical filaments, recognize conformational epitopes on recombinant tau. J. Neurosci. Res. *48*, 128–132. https://doi.org/10.1002/(sici)1097-4547(19970415)48:2%253C128::aid-jnr5%253E3.0.co;2-e.
33. Bonda, D.J., Smith, M.A., Perry, G., Lee, H.g., Wang, X., and Zhu, X. (2011). The mitochondrial dynamics of AD. Curr. Pharm. Des. *17*, 3374–3380. https://doi.org/10.2174/138161211798072562.
34. Chatzispyrou, I.A., Held, N.M., Mouchiroud, L., Auwerx, J., and Houtkooper, R.H. (2015). Tetracycline antibiotics impair mitochondrial function and its experimental use confounds research Iliana. Cancer Res. *75*, 4446–4449. https://doi.org/10.1007/s10549-015-3663-1.Progestin.

35. Bartels, T., De Schepper, S., and Hong, S. (2020). Microglia modulate neurodegeneration in Alzheimer's and Parkinson's diseases. Science *370*, 66–69. https://doi.org/10.1126/science.abb8587.

36. Heneka, M.T. (2019). Microglia take centre stage in neurodegenerative disease. Nat. Rev. Immunol. *19*, 79–80. https://doi.org/10.1038/s41577-018-0112-5.

37. Solito, E., and Sastre, M. (2012). Microglia function in Alzheimer's disease. Front. Pharmacol. *3*, 14. https://doi.org/10.3389/fphar.2012.00014.

38. Mathews, M., Wißfeld, J., Flitsch, L.J., Shahraz, A., Semkova, V., Breitkreuz, Y., Neumann, H., and Brüstle, O. (2023). Reenacting neuroectodermal exposure of hematopoietic progenitors enables scalable production of cryopreservable iPSC-derived human microglia. Stem Cell Rev. Rep. *19*, 455–474. https://doi.org/10.1007/s12015-022-10433-w.

39. Janda, E., Boi, L., and Carta, A.R. (2018). Microglial phagocytosis and its regulation: A therapeutic target in Parkinson's disease? Front. Mol. Neurosci. *11*, 144. https://doi.org/10.3389/fnmol.2018.00144.

40. Guan, Z., Chen, Z., Fu, S., Dai, L., and Shen, Y. (2020). Progranulin administration attenuates β-amyloid deposition in the hippocampus of 5xFAD mice through modulating BACE1 expression and microglial phagocytosis. Front. Cell. Neurosci. *14*, 260. https://doi.org/10.3389/fncel.2020.00260.

41. Collins, H.M., and Greenfield, S. (2024). Rodent models of Alzheimer's disease: Past misconceptions and future prospects. Int. J. Mol. Sci. *25*, 6222. https://doi.org/10.3390/ijms25116222.

42. Shi, Y., Kirwan, P., Smith, J., MacLean, G., Orkin, S.H., and Livesey, F.J. (2012). A human stem cell model of early Alzheimer's disease pathology in Down syndrome. Sci. Transl. Med. *4*, 124ra29. https://doi.org/10.1126/scitranslmed.3003771.

43. Hebisch, M., Klostermeier, S., Wolf, K., Boccaccini, A.R., Wolf, S.E., Tanzi, R.E., and Kim, D.Y. (2023). The impact of the cellular environment and aging on modeling Alzheimer's disease in 3D cell culture models. Adv. Sci. *10*, 2205037. https://doi.org/10.1002/advs.202205037.

44. Liedmann, A., Frech, S., Morgan, P.J., Rolfs, A., and Frech, M.J. (2012). Differentiation of human neural progenitor cells in functionalized hydrogel matrices. Biores. Open Access *1*, 16–24. https://doi.org/10.1089/biores.2012.0209.

45. Chandrasekaran, A., Avci, H.X., Ochalek, A., Rösingh, L.N., Molnár, K., László, L., Bellák, T., Téglási, A., Pesti, K., Mike, A., et al. (2017). Comparison of 2D and 3D neural induction methods for the generation of neural progenitor cells from human induced pluripotent stem cells. Stem Cell Res. *25*, 139–151. https://doi.org/10.1016/j.scr.2017.10.010.

46. Papadimitriou, C., Celikkaya, H., Cosacak, M.I., Mashkaryan, V., Bray, L., Bhattarai, P., Brandt, K., Hollak, H., Chen, X., He, S., et al. (2018). 3D culture method for Alzheimer's disease modeling reveals Interleukin-4 rescues Aβ42-induced loss of human neural stem cell plasticity. Dev. Cell *46*, 85–101.e8. https://doi.org/10.1016/j.devcel.2018.06.005.

47. Hurley, E.M., Mozolewski, P., Dobrowolski, R., and Hsieh, J. (2023). Familial Alzheimer's disease-associated PSEN1 mutations affect neurodevelopment through increased Notch signaling. Stem Cell Rep. *18*, 1516–1533. https://doi.org/10.1016/j.stemcr.2023.05.018.

48. Söllvander, S., Nikitidou, E., Brolin, R., Söderberg, L., Sehlin, D., Lannfelt, L., and Erlandsson, A. (2016). Accumulation of amyloid-β by astrocytes result in enlarged endosomes and microvesicle-induced apoptosis of neurons. Mol. Neurodegener. *11*, 38. https://doi.org/10.1186/s13024-016-0098-z.

49. Grathwohl, S.A., Kälin, R.E., Bolmont, T., Prokop, S., Winkelmann, G., Kaeser, S.A., Odenthal, J., Radde, R., Eldh, T., Gandy, S., et al. (2009). Formation and maintenance of Alzheimer's disease β-amyloid plaques in the absence of microglia. Nat. Neurosci. *12*, 1361–1363. https://doi.org/10.1038/nn.2432.

50. Gate, D., Rezai-Zadeh, K., Jodry, D., Rentsendorj, A., and Town, T. (2010). Macrophages in Alzheimer's disease: The blood-borne identity. J. Neural Transm. *117*, 961–970. https://doi.org/10.1007/s00702-010-0422-7.

51. Hansson, O., Lehmann, S., Otto, M., Zetterberg, H., and Lewczuk, P. (2019). Advantages and disadvantages of the use of the CSF Amyloid β (Aβ) 42/40 ratio in the diagnosis of Alzheimer's Disease. Alzheimers Res. Ther. *11*, 34. https://doi.org/10.1186/s13195-019-0485-0.

52. Kato, S., Gondo, T., Hoshii, Y., Takahashi, M., Yamada, M., and Ishihara, T. (1998). Confocal observation of senile plaques in Alzheimer's disease: Senile plaque morphology and relationship between senile plaques and astrocytes. Pathol. Int. *48*, 332–340. https://doi.org/10.1111/j.1440-1827.1998.tb03915.x.

53. Perl, D.P. (2010). Neuropathology of Alzheimer's Disease. Mt. Sinai J. Med. *77*, 32–42. https://doi.org/10.1002/msj.20157.Neuropathology.

54. Kwan, A.C., Duff, K., Gouras, G.K., and Webb, W.W. (2009). Optical visualization of Alzheimer's pathology via multiphoton-excited intrinsic fluorescence and second harmonic generation. Opt. Express *17*, 3679–3689. https://doi.org/10.1364/OE.17.003679.

55. Blazquez-Llorca, L., Garcia-Marin, V., and Defelipe, J. (2010). Pericellular innervation of neurons expressing abnormally hyperphosphorylated tau in the hippocampal formation of Alzheimer's disease patients. Front. Neuroanat. *4*, 20–23. https://doi.org/10.3389/fnana.2010.00020.

56. Mertens, J., Stüber, K., Poppe, D., Doerr, J., Ladewig, J., Brüstle, O., and Koch, P. (2013). Embryonic stem cell-based modeling of tau pathology in human neurons. Am. J. Pathol. *182*, 1769–1779. https://doi.org/10.1016/j.ajpath.2013.01.043.

57. Miguel, L., Rovelet-Lecrux, A., Feyeux, M., Frebourg, T., Nassoy, P., Campion, D., and Lecourtois, M. (2019). Detection of all adult Tau isoforms in a 3D culture model of iPSC-derived neurons. Stem Cell Res. *40*, 101541. https://doi.org/10.1016/j.scr.2019.101541.

58. Lovejoy, C., Alatza, A., Arber, C., Galasso, G., Bradshaw, T.Y., Verheyen, A., Lashley, T., Revesz, T., Hardy, J., Karch, C.M., and Wray, S. (2020). Engineered cerebral organoids recapitulate adult tau expression and disease-relevant changes in tau splicing. Research Square. https://doi.org/10.21203/rs.3.rs-37620/v1.

59. Kühlbrandt, W. (2015). Structure and function of mitochondrial membrane protein complexes. BMC Biol. *13*, 1–11. https://doi.org/10.1186/s12915-015-0201-x.

60. Yao, J., Irwin, R.W., Zhao, L., Nilsen, J., Hamilton, R.T., and Brinton, R.D. (2009). Mitochondrial bioenergetic deficit precedes Alzheimer's pathology in female mouse model of Alzheimer's disease. Proc. Natl. Acad. Sci. USA *106*, 14670–14675. https://doi.org/10.1073/pnas.0903563106.

61. Ryu, W.-I., Bormann, M.K., Shen, M., Kim, D., Forester, B., Park, Y., So, J., Seo, H., Sonntag, K.-C., and Cohen, B.M. (2021). Brain cells derived from Alzheimer's disease patients have multiple specific innate abnormalities in energy metabolism. Mol. Psychiatry *26*, 5702–5714. https://doi.org/10.1038/s41380-021-01068-3.

62. Wee, M., Chegini, F., Power, J.H.T., and Majd, S. (2018). Tau positive neurons show marked mitochondrial loss and nuclear degradation in Alzheimer's disease. Curr. Alzheimer Res. *15*, 928–937. https://doi.org/10.2174/1567205015666180613115644.

63. Sasaguri, H., Nilsson, P., Hashimoto, S., Nagata, K., Saito, T., De Strooper, B., Hardy, J., Vassar, R., Winblad, B., and Saido, T.C. (2017). APP mouse models for Alzheimer's disease preclinical studies. EMBO J. *36*, 2473–2487. https://doi.org/10.15252/embj.201797397.

64. Schoch, K.M., DeVos, S.L., Miller, R.L., Chun, S.J., Norrbom, M., Wozniak, D.F., Dawson, H.N., Bennett, C.F., Rigo, F., and Miller, T.M. (2016). Increased 4R-tau induces pathological changes in a human-tau mouse model. Neuron *90*, 941–947. https://doi.org/10.1016/j.neuron.2016.04.042.

65. Shi, Y., Kirwan, P., Smith, J., Robinson, H.P.C., and Livesey, F.J. (2012). Human cerebral cortex development from pluripotent stem cells to functional excitatory synapses. Nat. Neurosci. *15*, 477–486. https://doi.org/10.1038/nn.3041.

66. Sun, Z., Kwon, J.-S., Ren, Y., Chen, S., Walker, C.K., Lu, X., Cates, K., Karahan, H., Sviben, S., Fitzpatrick, J.A.J., et al. (2024). Modeling late-onset

Alzheimer's disease neuropathology via direct neuronal reprogramming. Science *385*, adl2992. https://doi.org/10.1126/science.adl2992.

67. Vermezovic, J., Adamowicz, M., Santarpia, L., Rustighi, A., Forcato, M., Lucano, C., Massimiliano, L., Costanzo, V., Bicciato, S., Del Sal, G., and d'Adda di Fagagna, F. (2015). Notch is a direct negative regulator of the DNA-damage response. Nat. Struct. Mol. Biol. *22*, 417–424. https://doi.org/10.1038/nsmb.3013.

68. Gallo, L.H., Akanda, N., Autar, K., Patel, A., Cox, I., Powell, H.A., Grillo, M., Barakat, N., Morgan, D., Guo, X., and Hickman, J.J. (2024). A functional aged human iPSC-cortical neuron model recapitulates Alzheimer's disease, senescence, and the response to therapeutics. Alzheimer's Dement. *20*, 5940–5960. https://doi.org/10.1002/alz.14044.

69. Chen, G., Gulbranson, D.R., Hou, Z., Bolin, J.M., Ruotti, V., Probasco, M.D., Smuga-Otto, K., Howden, S.E., Diol, N.R., Propson, N.E., et al. (2011). Chemically defined conditions for human iPSC derivation and culture. Nat. Methods *8*, 424–429. https://doi.org/10.1038/nmeth.1593.

70. Koch, P., Breuer, P., Peitz, M., Jungverdorben, J., Kesavan, J., Poppe, D., Doerr, J., Ladewig, J., Mertens, J., Tüting, T., et al. (2011). Excitation-induced ataxin-3 aggregation in neurons from patients with Machado–Joseph disease. Nature *480*, 543–546. https://doi.org/10.1038/nature10671.

71. Roese-Koerner, B., Stappert, L., Berger, T., Braun, N.C., Veltel, M., Jungverdorben, J., Evert, B.O., Peitz, M., Borghese, L., and Brüstle, O. (2016). Reciprocal regulation between bifunctional miR-9/9* and its transcriptional modulator Notch in human neural stem cell self-renewal and differentiation. Stem Cell Rep. *7*, 207–219. https://doi.org/10.1016/j.stemcr.2016.06.008.

72. Meijering, E., Jacob, M., Sarria, J.C.F., Steiner, P., Hirling, H., and Unser, M. (2004). Design and validation of a tool for neurite tracing and analysis in fluorescence microscopy images. Cytometry *58*, 167–176. https://doi.org/10.1002/cyto.a.20022.

73. Preibisch, S., Saalfeld, S., and Tomancak, P. (2009). Globally optimal stitching of tiled 3D microscopic image acquisitions. Bioinformatics *25*, 1463–1465. https://doi.org/10.1093/bioinformatics/btp184.

74. Wischhof, L., Lee, H.-M., Tutas, J., Overkott, C., Tedt, E., Stork, M., Peitz, M., Brüstle, O., Ulas, T., Händler, K., et al. (2022). BCL7A-containing SWI/SNF/BAF complexes modulate mitochondrial bioenergetics during neural progenitor differentiation. EMBO J. *41*, e110595. https://doi.org/10.15252/embj.2022110595.

75. Connolly, N.M.C., Theurey, P., Adam-Vizi, V., Bazan, N.G., Bernardi, P., Bolaños, J.P., Culmsee, C., Dawson, V.L., Deshmukh, M., Duchen, M.R., et al. (2018). Guidelines on experimental methods to assess mitochondrial dysfunction in cellular models of neurodegenerative diseases. Cell Death Differ. *25*, 542–572. https://doi.org/10.1038/s41418-017-0020-4.

STAR★METHODS

KEY RESOURCES TABLE

REAGENT or RESOURCE	SOURCE	IDENTIFIER
**Antibodies**		
Mouse purified anti-β-Amyloid	BioLegend	Cat# SIG-39320; RRID:AB_2564652
Rabbit oligomer A11 polyclonal Antibody	ThermoFisher	Cat# AHB0052; RRID:AB_10376183
Mouse Phospho-Tau (Ser202, Thr205) Monoclonal Antibody (AT8)	ThermoFisher	Cat# MN1020; RRID:AB_223647
Mouse Phospho-Tau (Thr181) Monoclonal Antibody (AT270)	ThermoFisher	Cat# MN1050; RRID:AB_223651
Rabbit cCas3 Antibody	Promega	N/A
Mouse Anti-Complex IV Immunocapture antibody	Abcam	N/A
Rabbit beta-Amyloid (D54D2) Monoclonal Antibody	Cell Signaling	Cat# 8243; RRID:AB_2797642
Rabbit Dach1 Polyclonal Antibody	Proteintech	Cat# 10914-1-AP; RRID:AB_2230330
Rabbit GABA Antibody	Sigma	N/A
Rabbit Anti-GFAP Antibody	Millipore	N/A
Rabbit Phospho-Histone H2A.X (Ser139) (20E3) Monoclonal Antibody	Cell Signaling	Cat# 9718; RRID:AB_2118009
Rabbit IBA1 Monoclonal Antibody	Synaptic Systems	N/A
Mouse MC-1 Antibody	Peter Davies	N/A
Rabbit Nestin Antibody	Novus Biologicals	N/A
Rabbit Anti-Neurofilament Antibody	Abcam	N/A
Rabbit PAX6 Antibody	Covance	N/A
Mouse PLZF Antibody	R&D Systems	N/A
Mouse S100 Protein Monoclonal Antibody	ThermoFisher	N/A
Mouse SOX2 Antibody	R&D Systems	N/A
Mouse SSEA Monoclonal Antibody	DSHB	N/A
Mouse Anti-TOM20 Antibody	Abcam	N/A
Total OXPHOS Rodent WB Antibody Cocktail	Abcam	Cat# ab110413; RRID:AB_2629281
Mouse Anti-TRA-1-81	Millipore	Cat# MAB4381; RRID:AB_177638
Mouse anti-Tubulin β 3 (TUBB3) Antibody	Covance	N/A
Rabbit Monoclonal VGluT1 Antibody	Abcam	Cat# ab227805; RRID:AB_2868428
Rabbit ZO-1 Polyclonal Antibody	Invitrogen	Cat# 13462987; RRID:AB_2546345
**Experimental models: Cell lines**		
ILB-C14m-s11 iPSCs	Universitätsklinikum Bonn (UKB)	UKBi017-A
**Oligonucleotides**		
AAVS1 5' sequencing primer fw ACCAACGCCGACGGTATCAG	Institute for Reconstructive Neurobiology, Bonn	N/A
AAVS1 5′ sequencing primer rv1 CAGACCCTTGCCCTGGTGGT	Institute for Reconstructive Neurobiology, Bonn	N/A
AAVS1 5′ sequencing primer rv2 CACCAGGATCAGTGAAACGC	Institute for Reconstructive Neurobiology, Bonn	N/A
AAVS1 3′ sequencing primer fw TACCACCGATTCTATGCCCC	Institute for Reconstructive Neurobiology, Bonn	N/A
AAVS1 3′ sequencing primer rv AGGATGCAGGACGAGAAACA	Institute for Reconstructive Neurobiology, Bonn	N/A
**Recombinant DNA**		
AAVS1-APPSwe/Lon-PSEN1dE9 plasmid	Institute for Reconstructive Neurobiology, Bonn	N/A

CellPress
OPEN ACCESS

EXPERIMENTAL MODEL AND STUDY PARTICIPANT DETAILS

IPSC culture and quality control

ILB-C14m-s11 iPSCs (https://hpscreg.eu/cell-line/UKBi017-A) originally generated from human skin fibroblasts of a male donor via Sendai virus transduction with four reprogramming factors were maintained in E8 medium for AAVS1 targeting and later in StemMACS iPS-Brew XF medium (Miltenyi Biotec) on Geltrex-coated (1:90, ThermoFisher) dishes in a 37°C incubator at 5% CO_2.[69] The cells grew as colonies until close to confluency with medium replacement every other day and were passaged using PBS-EDTA. Pluripotency of the iPSC line was confirmed previously by teratoma assay, as described in Koch et al.[70] The studies were conducted in accordance with the legal requirements of the local authorities (permit number: AZ 8.87–50.10.37.09.290). Cells were regularly tested for mycoplasma contamination via PCR. The use of these lines was approved by the Ethics Committee of the Medical Faculty of the University of Bonn (approval number 275/08), and informed written consent was obtained from the donors. All experiments were performed in accordance with German guidelines and regulations.

METHOD DETAILS

Karyotyping

Genomic integrity of hiPSCs was validated by array-based single-nucleotide polymorphism (SNP) analysis for the parental hiPSC line and for each clonal line established after AAVS1 targeting at the Institute of Human Genetics, University of Bonn.

Generation of triple-mutant iPSCs

The AAVS1-GFP targeting plasmid and the corresponding TALENs were provided by Su-Chun Zhang, Sanford Burnham Prebys. Plasmid sequencing was performed by a commercial sequencing service.

IPSCs were nucleofected using the Amaxa nucleofector 2 (Lonza) with cell line kit V (Lonza). Cultures were pre-treated with 10 μM Y-27632 (Biotechne) one hour before dislodgement by PBS-EDTA and centrifugation. The pellet was then resuspended in 100 μL of nucleofection mix, transferred into a nucleofection cuvette and nucleofected with program B-023. Afterward, the cell suspension was collected from the cuvette and distributed on a Geltrex-coated (1:90) dish with StemMACS iPS-Brew XF supplemented with 10 μM Y-27632 and 5 μM L755507 (Sigma-Aldrich). During the first 72 h, medium was replaced daily with Stembrew plus 10 μM Y-27632. Subsequently, 0.3 μg/mL puromycin (Sigma-Aldrich) was added during daily medium changes for 5–14 days. Upon formation of colonies with diameters >500 μm, colonies were partially harvested. One-half of a colony was transferred to a Geltrex-coated (1:90) 96-well plate, the other half used for genotyping. Picked colonies were expanded, karyotyped and cryopreserved.

For PCR-based genotyping, puromycin-resistant colonies were picked from primary plates. Approximately half of each colony was transferred into 30 μL of QuickExtract reagent (Lucigen). Transgene integration was validated by multiplex PCR for the 5′ end of the AAVS1 locus (fw: 5-ACCAACGCCGACGGTATCAG-3; rv1: 5-CAGACCCTTGCCCTGGTGGT-3; rv2: 5-CACCAGGATCAGTGAA ACGC-3) and regular PCR for the 3′ end (fw: 5-TACCACCGATTCTATGCCCC-3, rv: 5-AGGATGCAGGACGAGAAACA-3).

Generation of 3D cultures

Lt-NES cells were derived from iPSCs as described previously by Koch et al.[21] with modifications from Roese-Koerner et al.[71] Cells were propagated in lt-NES cell medium (DMEM/F12, 1% N2 supplement, 1.6 mg/mL glucose, 10 ng/mL bFGF, 10 ng/mL EGF, 0.1% B27). Cultures were passaged with trypsin in a 1:2-1:3 ratio every 2–3 days. For neuronal differentiation, lt-NES cells were seeded on Geltrex-coated dishes in NGM medium (48.75% Neurobasal medium, 48.75% N2 medium, 2% B27, 0.5% 100 mM glutamine).

3D cultures were prepared as previously described.[23] Thin-layer cultures for imaging were generated by resuspending 2×10^5 lt-NES cells per well of a 96-well in 100 μL ice-cold lt-NES medium containing 10% Geltrex. Then, the suspension was dispensed into an imaging-grade, flat-bottom 96-well plate (μClear, Greiner) and left to solidify for 30 min in an incubator at 37°C incubator and 5% CO_2. Finally, 100 μL lt-NES medium was added that was replaced with NGM on the next day. To generate thick-layer cultures for protein analyses, 4×10^6 lt-NES cells per 24-well transwell insert were embedded in 200 μL of a 50% Geltrex, 50% lt-NES medium suspension. After one hour at 37°C, the outer wells were filled up with lt-NES medium that was replaced with NGM on the next day. On day 10, 3D cultures were either cultivated using NGM, NGM plus doxycycline (1 μg/mL, Sigma-Aldrich) or NGM plus doxycycline (1 μg/mL, Sigma-Aldrich) and γ-secretase inhibitor (GSI) (DAPT, 10 μM, Axon MedChem or RO4929097, 100 nM, Roche) for the remainder of the experiment. Medium was replaced every other day; cultures were maintained for 8 to 14 weeks. Long-term stability of transgene expression from the AAVS1 locus was confirmed by robust mCherry expression up to 14 weeks of Dox treatment (see also Figure S3H). Cell-free thin layer matrices were generated using 100 μL lt-NES medium containing 10% Geltrex per well of a 96-well (μClear, Greiner). Medium changes were carried out according to the protocol as described above.

Immunofluorescence analyses

All immunostained cultures were fixed with 4% para-formaldehyde in PBS over night at room temperature and treated with a combined blocking and permeabilization solution (5% FCS, 0.1% Triton X-100 in PBS) for 1 h at RT. For GABA staining, 4% PFA plus 0.02% glutaraldehyde was used. Primary antibodies were applied in blocking solution over night at 4°C and cultures were washed

3 × 5 min with PBS. Secondary antibodies were incubated for 1–4 h at RT and washed 3 × 5 min with PBS. Thick-layer cultures were stained analogously, however, blocking, primary and secondary antibody incubation steps were extended to overnight at 4°C.

Imaging of amyloid and p-tau deposition

All amyloid dyes were employed on PFA-fixed cultures. A 5 mM Thioflavin T (ThT, Sigma-Aldrich) solution was prepared in H_2O and sterile filtered as 1000x stock. Staining was performed using 5 μM ThT solution for 8 min at room temperature followed by several rinses with 1:1 ethanol in PBS and one final PBS wash. A 1.5 mM Methoxy-X04 (Cayman) solution was prepared in 1:1:2 H_2O:ethanol:DMSO and diluted 1:100 in 1:1 ethanol:H_2O prior to staining. Samples were incubated with M-X04 solution for 30 min at room temperature, rinsed twice with 80% ethanol in H_2O and several times with PBS. Congo red solutions were prepared as described.[29] Samples were incubated with alcoholic salt solution for 20 min at room temperature, 20 min with Congo red staining solution at room temperature and rinsed repeatedly with PBS. Amytracker 630 (Ebba Biotech) was diluted 1:500 in PBS, applied to samples for 30 min at room temperature and rinsed repeatedly with PBS.

Imaging locations were chosen randomly within each well. Imaging was performed at intermediate depths as computed based on the topmost and bottommost cells at each location in the 3D matrix. Regions with gel matrix disruptions were excluded. Due to the difficulty of acquiring accurate cell counts in 3D cultures and limitations in channel availability due to the presence of fluorescent reporters, marker validation stainings were normalized to culture area rather than cell number. Neurites were traced using the ImageJ/FIJI plugin NeuronJ in single-plane confocal images.[72] Only AT8-positive cell process segments were traced.

Area fluorescence was determined from the combined pixel intensities of stitched images covering a complete well of a 96-well plate (IN Cell Analyzer, GE Healthcare, 4 × 4 images, 10x objective). Images were non-overlapping and not adjusted for brightness during stitching. Stitching was done using the Grid/Collection stitching plugin for ImageJ/FIJI.[73]

Quantification of Aβ species

Supernatants of 2D and (thick-layer) 3D cultures were collected by aspiration from the live culture after incubating for 24–48 h. Unless stated otherwise, cultures were incubated with 2 mL of NGM medium and, where appropriate, Dox or Dox+GSI. Approximately 1.5 mL supernatant was collected and centrifuged at 16,000 g for 10 min to remove particulate matter. Supernatant samples for ELISA were collected from differentiated 2D $APP_{Swe/Lon\text{-}PSEN1\Delta E9}$ cultures after 4–8 weeks of treatment. The crude lysates were cleared by filtration through a 0.4 μm sterile filter unit and subjected to ELISA analysis using the "Human β Amyloid (1–40) ELISA Kit Wako II" and "Human β Amyloid (1–42) ELISA Kit Wako, High Sensitivity" (FUJIFILM) according to the manufacturer's instructions. The induced supernatants were diluted 1:10 and 1:100, respectively, prior to measurement. For microglial co-cultures, cell-free supernatant samples were harvested after 57 days of cultivation, and the cells embedded in Geltrex were collected by dissociation in 250 μL RIPA buffer (Sigma) containing 2.5 μL of protease and phosphatase inhibitor (Thermo Fischer) for 30 min on ice and subsequent centrifugation at 10,000 g for 10 min at 4°C. Samples were stored at −80°C before measuring Aβ40 and Aβ42 concentrations using the V-PLEX Aβ Peptide Panel (6E10) Kit (Meso Scale Discovery) on the Meso Scale Discovery QuickPlex SQ120 system, according to the manufacturer's protocols.

Western blotting

Protein extraction, supernatant collection and western blotting were performed as previously described.[23] Briefly, 3D cultures were collected by mechanical detachment followed by centrifugation to separate the cells and matrix from remaining supernatant. For solubility fractionation, the culture pellets were sequentially homogenized in TBS and SDS buffers and formic acid, and the insoluble fractions were collected after pelleting at 100,000 g during each step. For the evaluation of total tau (t-tau) and phospho tau (p-tau) Ser202/Thr205 levels, a self-made RIPA buffer (250 mM Tris-HCl, 5 mM EDTA, 750 mM NaCl, 5% Igepal, 2.5% deoxycholic acid, 0.5% SDS) was used to collect the 3D cultures, followed by a harshly pipetting for 30′ keeping the tube on ice and centrifugation at 10,000 g for 10 min at 4°C. The pellet was discarded, while the supernatant was processed further. Total tau and p-tau were detected with the following antibodies respectively: mouse tau antibody (Thermo Fisher Scientific) and mouse Phospho-Tau Ser202, Thr205 (Thermo Fisher Scientific). Supernatants from 2D and 3D cultures were collected from cultures seeded with 2 million cells after incubation with 4 mL medium for 24 h. Samples were separated on either 12% or 4–12% Bis-Tris gels for western blot analysis as described in the respective experiment. Mitochondrial proteins were detected with the following antibodies: Total OXPHOS Rodent WB Antibody Cocktail (abcam), mouse α-MT COIV (abcam), mouse α-β-actin (Sigma) and HRP-conjugated goat α-mouse (Thermo Fisher Scientific).

Mitochondrial analysis

Mitochondrial network size was quantified in 3D cultures after immunolabeling with antibodies against neurofilament (heavy chain), p-tau (AT8) and mitochondria (TOM20). Using confocal z-stacks, single cells were identified and three-dimensionally masked based on the somatic segment of the neurofilament staining. Using this mask, the intracellular mitochondrial network was reconstructed as a three-dimensionally branching tube network. The mitochondrial network size was defined as the longest non-branching and non-crossing path that can be traced across each network.[74] To determine oxygen consumption rate (OCR), 3D cultures were set up in Seahorse XF 24-well plates (Agilent) using the thin-layer protocol.[75] OCR measurements were performed after 8 weeks of differentiation in total and 6 weeks of Dox-treatment, respectively. Before measurement, cultures were equilibrated to atmospheric CO_2 for

CellPress
OPEN ACCESS

1 h at 37°C in NGM. The analysis was performed with 8 time points per treatment condition according to the manufacturer's instructions in response to 2 μM oligomycin, 2 μM FCCP and 1 μM rotenone and antimycin.

Microglia co-culture

IPSC line iLB-C133bm-S4, cultured as described above, was differentiated into iPSC-derived microglia (iPSdMiG) according to a previously published protocol.[38] IPSdMiG were directly seeded onto 52-day-old lt-NES cell-containing thin layer cultures (or acellular matrices where indicated) at a density of 2×10^4 cells per well of a 96-well-plate (μClear, Greiner). Specifically, the cell culture medium was removed and replaced by NGM media containing iPSdMiG and supplemented with 100 ng/mL MCSF (Peprotech). Cells were left to invade the matrix before the next media change after 24 h. The second media change was performed after 96 h. The analysis was conducted after 5 days of cocultivation.

For microglial co-cultures, imaging locations were chosen and analyzed as described above. Quantification of cleaved caspase 3 (cCas3) and 6E10 phagocytosis was performed using custom ImageJ macros. Confocal Z-Stacks and three-dimensional reconstruction were used to probe whether aggregated 6E10 and cCas3-positive nuclei were located inside the iPSdMiG.

QUANTIFICATION AND STATISTICAL ANALYSIS

Statistical analysis

Statistical analysis was performed either using R (ANOVA, Kruskal-Wallis), GraphPad Prism (ANOVA) or Microsoft Excel (Student's *t* test). ANOVA indicates a one-way ANOVA with Tukey's post-hoc test. Kruskal-Wallis was employed for non-parametric analysis of non-normally distributed measurements in conjunction with Nemenyi's post-hoc test. Student's *t* test was used for hypothesis-driven (i.e., one-tailed) dual comparisons of unpaired samples unless stated otherwise. Seahorse experiments were analyzed by averaging the measurements from all time points between compound additions as technical repeats, for each cell line and experiment, followed by Kruskal-Wallis testing. Detailed information can be found in the corresponding figure legends.

Cell Reports Methods, Volume *6*

Supplemental information

Recapitulation of plaque formation, tau pathology, and neurodegeneration in a human 3D matrix model of Alzheimer's disease

Matthias Hebisch, Viola Kamin, Giovanna Cenini, Antonia Piazzesi, Fabio Bertan, Beatrice Weykopf, Julia Schlee, Senthilvelrajan Kaniyappan, Kevin J. Washicosky, Doo Yeon Kim, Daniele Bano, Michael Peitz, and Oliver Brüstle

Supplemental Figure 1

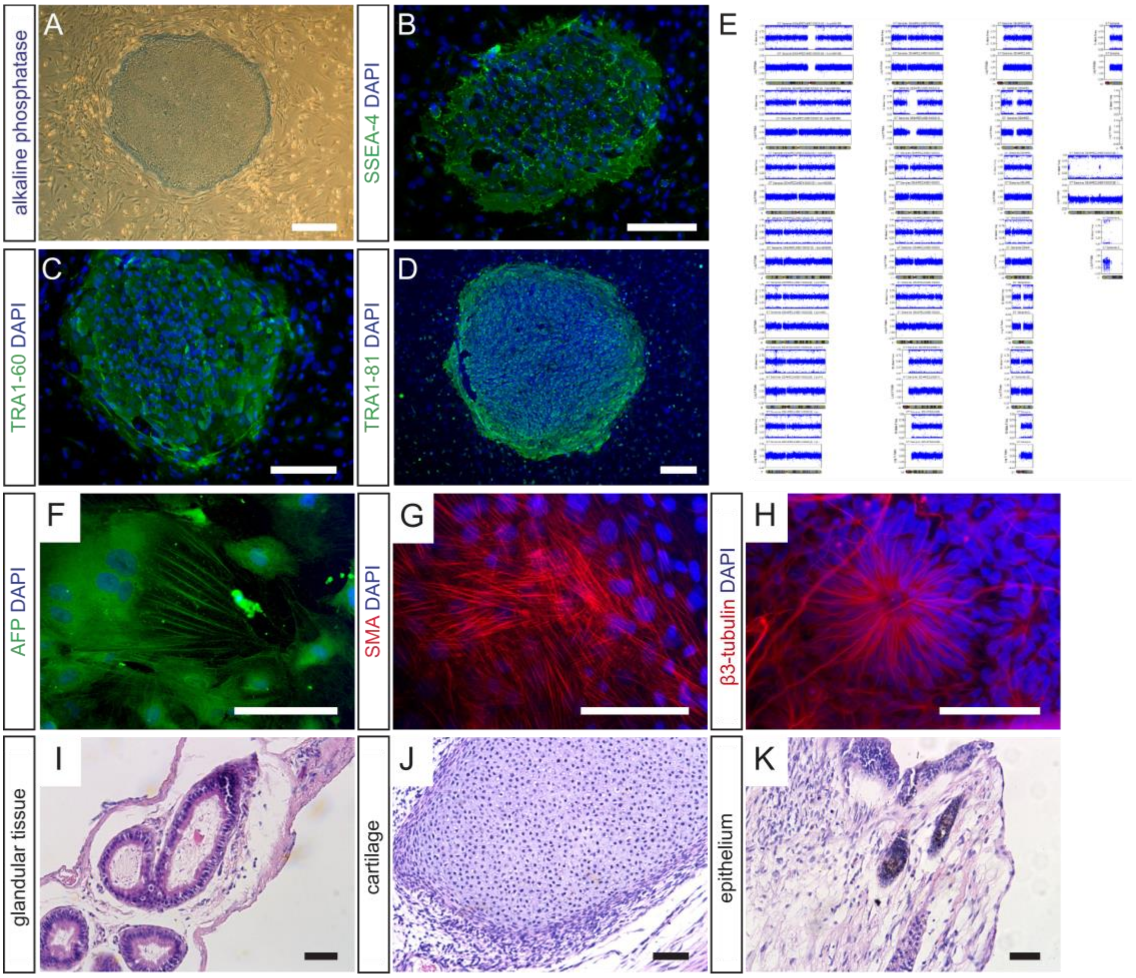

Parental iPS cell line, related to Figure 1

Expression of typical pluripotency markers in iPSC clone iLB-C14m-s11 was validated by immunohistochemistry for expression of (**A**) alkaline phosphatase, (**B**) SSEA4, (**C**) TRA-1-60 and (**D**) TRA-1-81. Scale bars = 100 µm. (**E**) Genomic integrity analysis of the iLB-C14m-s11 iPSC line via SNP panel. Differentiation potential was shown *in vitro* ((**F**) AFP (alpha-fetoprotein, marks endoderm), (**G**) SMA (smooth muscle actin, marks mesoderm), (**H**) β3-tubulin (marks ectoderm)) and *in vivo* via teratoma formation ((**I**) glandular tissue (endoderm), (**J**) cartilage (mesoderm) and (**K**) pigment epithelium (ectoderm)). Scale bar = 100 µm.

Supplemental Figure 2

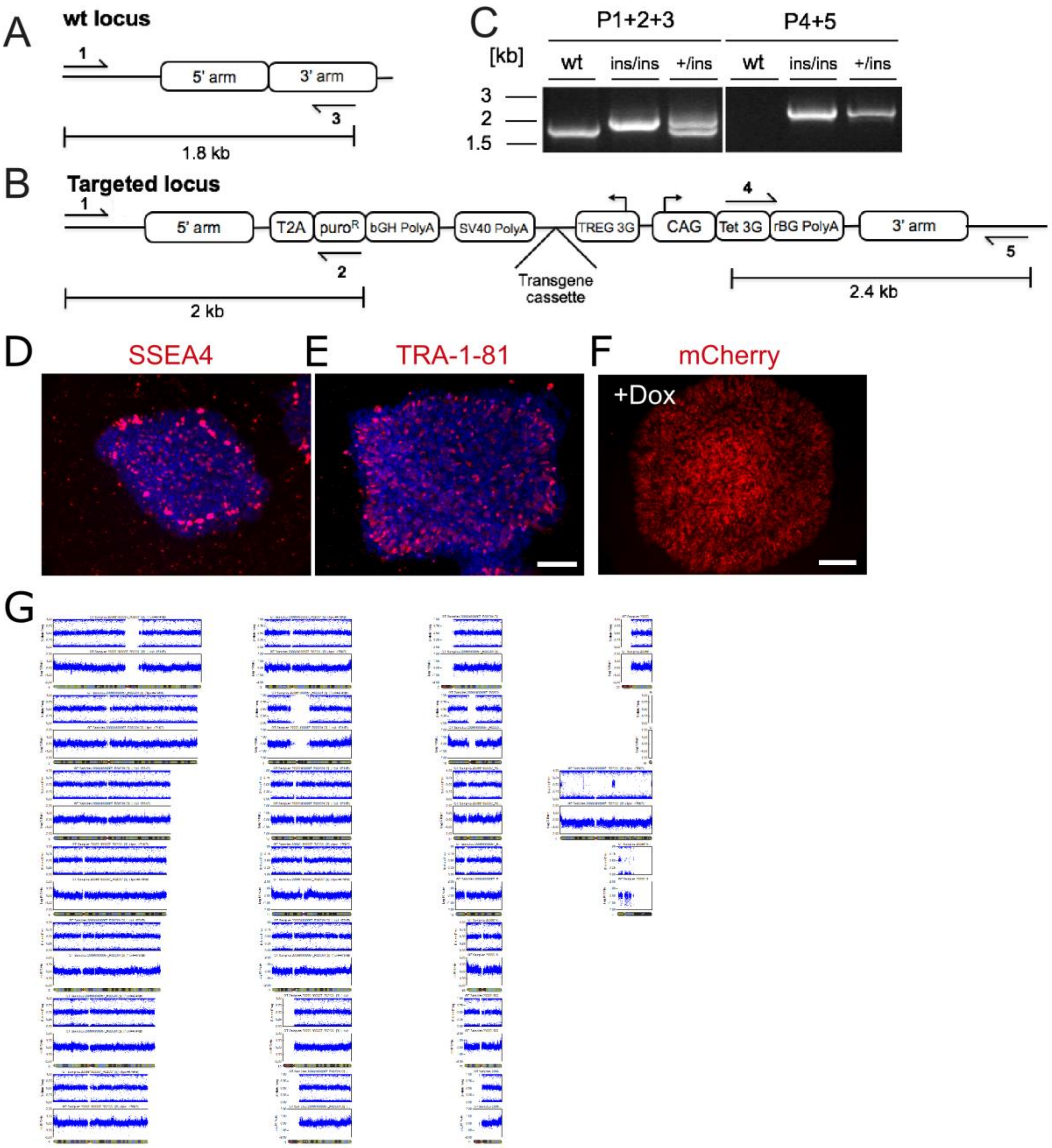

Validation of AAVS1 safe harbor transgene insertion, related to Figure 1

(**A**) Multiplex PCR using 3 primers for single-reaction analysis of 5‘ AAVS1 targeting in iPS colony samples of approximately 200-500 cells. Primer binding on untargeted alleles yields 1.8 kb products, whereas (**B**) binding outside the target region and inside the puromycin resistance gene yields a 2 kb product. A P4+5 pairing confirms the 3’ integration of the transgene cassette by generating a 2.4 kb product. (**C**) AAVS1-*APP*$_{Swe/Lon}$-*PSEN1ΔE9* genotyping in wild-type, homozygously and heterozygously integrated iPS cell clones. (**D,E**) Immunofluorescence detection of pluripotency-associated cell-surface

markers SSEA4 and TRA-1-81 (both red) in homozygous AAVS1-*APP*$_{Swe/Lon}$-*PSEN1ΔE9* iPS cell clones. Nuclear DAPI stain (blue). Scale bar = 100 µm. (**F**) Detection of the mCherry fluorescent marker in homozygous AAVS1-*APP*$_{Swe/Lon}$-*PSEN1ΔE9* iPS cells after 72 h of doxycycline induction. Scale bar = 200 µm. (**G**) Genomic integrity of homozygous AAVS1-APP$_{Swe/Lon}$-PSEN1ΔE9 iPS cells was assessed by SNP genotyping. For each chromosome the B allele frequency (upper row) and the log R ratio (lower row) are shown. No major genomic alterations could be detected.

Supplemental Figure 3

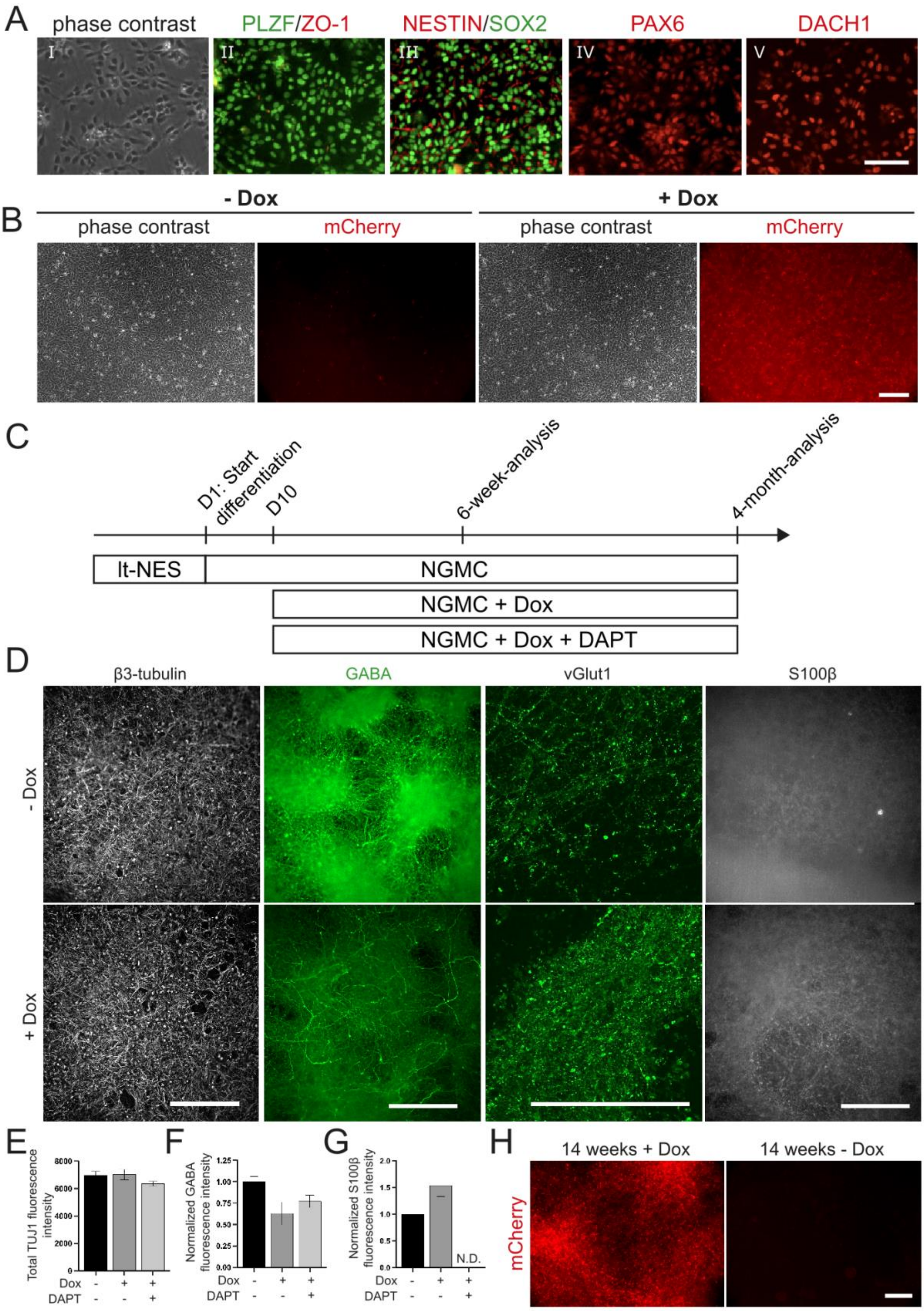

Validation of neural stem cells and neuronal cultures, related to Figure 1

(**A**) *$APP_{Swe/Lon\text{-}PSEN1\Delta E9}$* lt-NES cells exhibit a rosette-like growth pattern (phase contrast) and expression of the neural stem cell markers PLZF, ZO-1, NESTIN, SOX2, PAX6 and DACH1. Scale bar = 100 µm. (**B**) Dox-induced expression of the mCherry reporter in *$APP_{Swe/Lon\text{-}PSEN1\Delta E9}$* lt-NES cells. Scale bar = 200 µm. (**C**) Schematic experimental outline. Lt-NES cells are embedded in a 3D matrix and differentiated for 10 days prior to application of the experimental condition to avoid transgene interference with early differentiation. Cultures were analyzed after 6 weeks or 4 months of treatment. (**D**) Representative micrographs of high-density 3D homozygous *$APP_{Swe/Lon\text{-}PSEN1\Delta E9}$* neurons stained for β3-tubulin, GABA, vGlut1 and S100β after 6 weeks of growth-factor withdrawal with and without doxycycline treatment. Scale bars = 500 µm. (**E-G**) Quantification of neural markers β3-tubulin (E), GABA (F), astrocyte marker S100β (G) in *$APP_{Swe/Lon\text{-}PSEN1\Delta E9}$* lt-NES differentiated for 6 weeks ± Dox and DAPT treatment (n=3; Student's t-test, one-tailed, unpaired). (**H**) Validation of transgene induction in homozygous *$APP_{Swe/Lon\text{-}PSEN1\Delta E9}$* neurons after 14 weeks of differentiation in the presence and absence of doxycycline (1 µg/ml, mCherry in red). All images from high-density areas. Scale bar = 200 µm. All data presented as mean ± SEM.

Supplemental Figure 4

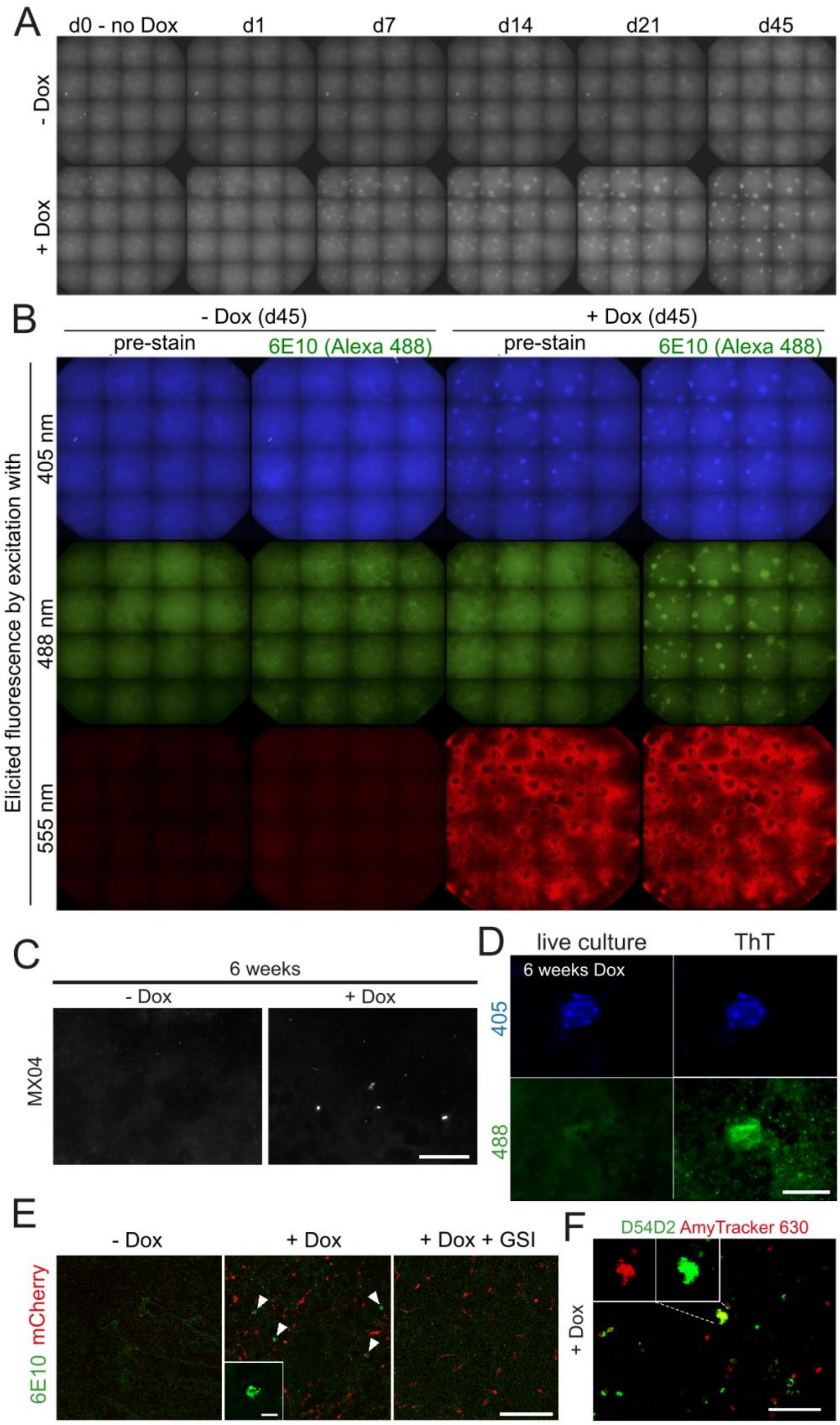

Autofluorescent amyloid deposits and dye-based stainings, related to Figure 2

(**A**) Stiched longitudinal overview of 96-well 3D cultures. Images show autofluorescence in the DAPI channel elicited by a 405 nm laser. (**B**) Stitched overview of 96-well 3D cultures from (A). Rows show fluorescence collected in the DAPI, GFP, and RFP channels upon excitation with 405 nm, 488 nm, and 555 nm, respectively. Pre-stain indicates fluorescence collected prior to antibody staining. 6E10 indicates fluorescence after antibody staining with 6E10 and secondary staining with an Alexa 488-tagged antibody. (**C**) Low magnification MX04 staining of 6-week-old cultures with and without doxycycline induction. MX04 fluorescence is depicted in greyscale (n=3). Scale bar = 200 µm. (**D**) Aggregate in a 6-week Dox-induced 3D culture. Autofluorescence elicited by 405 nm (emission in blue), and 488 nm (emission in green) light in a live culture and after PFA fixation and staining with ThT. Scale bar = 100 µm. (**E**) Detection of Aβ (6E10) and mCherry in 6-week Dox-induced, non-induced and induced GSI-treated 3D cultures. Confocal imaging; arrowheads indicate 6E10 accumulations. Scale bar = 100 µm, zoom-in: 10 µm. (**F**) Confocal micrograph of an amyloid aggregate with amyloid-β-specific antibody D54D2 (green) and amyloid fibril dye Amytracker 630 (red) in a 6-week Dox-induced culture. Scale bar = 50 µm.

Supplemental Figure 5

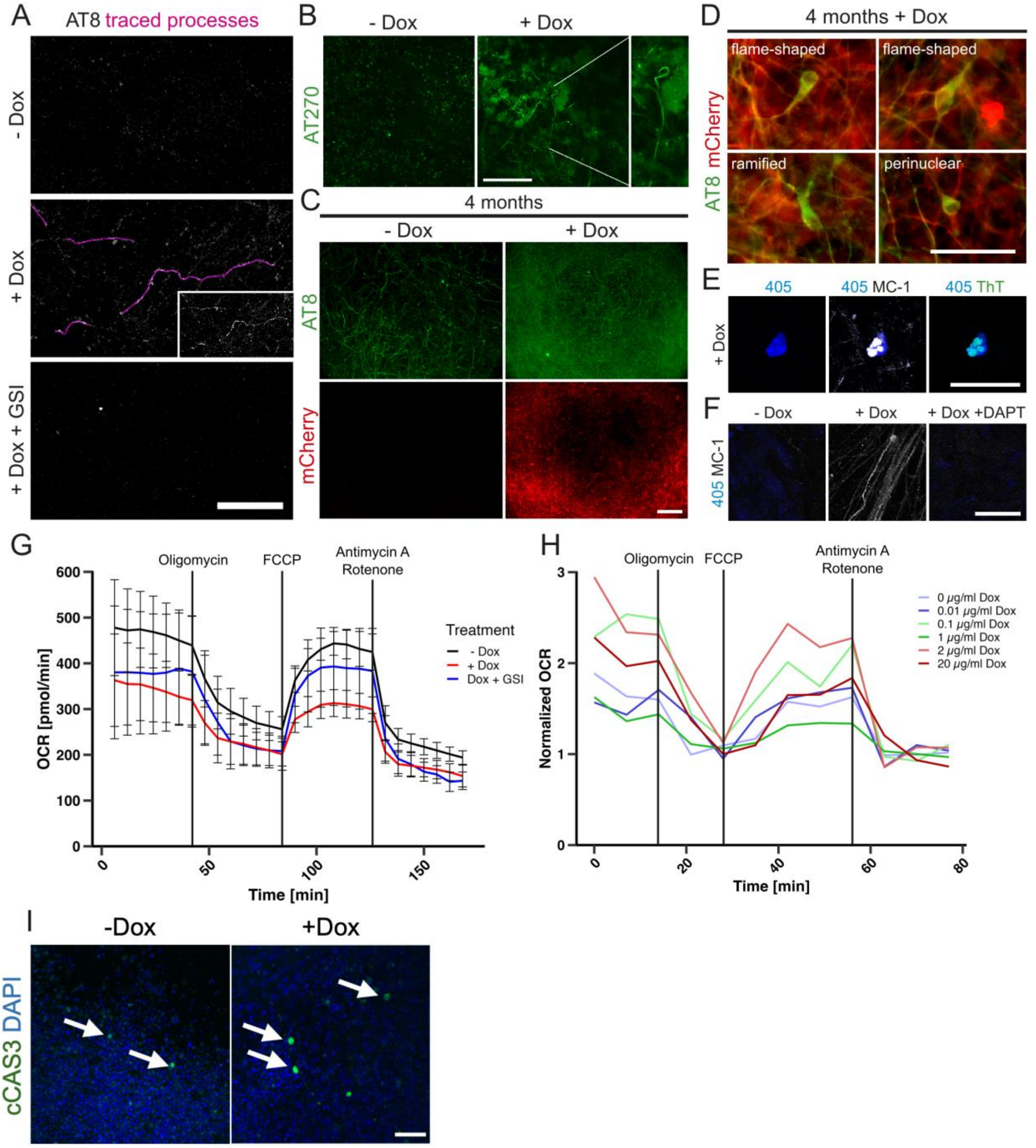

Tau pathology in *APP$_{Swe/Lon}$*, *PSEN1(ΔE9)* matrix cultures and assessment of mitochondrial respiration, related to Figures 3 and 4

(**A**) Illustration of traced AT8-positive neurites. Please note that the +Dox panel is also depicted as main Figure 3B. Scale bar = 100 µm. (**B**) 6-week Dox-induced cultures stained with the PHF antibody AT270. Magnification of a neuron with intraneuritic p-tau accumulation. Scale bar = 100 µm. (**C**) Example of a 4-month Dox-induced culture depicting anti-phospho-tau immunofluorescence (AT8, green) and mCherry (red). Scale bar = 200 µm. (**D**) High-magnification images of single 4-month Dox-induced neurons from (B)

showing p-tau accumulation and morphological alterations reminiscent of neurofibrillary tangles. Scale bar = 40 μm. (**E**) Aggregated p-tau in the “paperclip” conformation is a hallmark of tau pathology and neurofibrillary tangles. Detection of amyloid autofluorescence (blue), “paperclip” tau (MC-1 antibody, grayscale) and Thioflavin T (green) in 4-month Dox-induced cultures. Scale bar = 50 μm. (**F**) 4-months-induced cultures co-treated with 10 μM DAPT. Scale bar = 50 μm. (**G**) Oxygen consumption rate (OCR) of 6-week-induced lt-NES derived $APP_{Swe/Lon}$-*PSEN1ΔE9* neurons without induction (NGMC), treatment with Dox or Dox + the γ-secretase inhibitor DAPT (n=3; 6 technical replicates each). (**H**) Three weeks pre-differentiated, non-transgenic lt-NES-derived neurons were thawed and treated with 0.01 - 20 μg/ml doxycycline for 1 week before Seahorse measurement. Data were stratified into control (0), low (0.01, 0.1), medium (1, 2) and high (20) doxycycline concentrations for clarity (Lines represent mean values: 3 technical replicates per condition). (**I**) Cleaved caspase-3 staining of 6-week-induced 3D cultures with nuclear stain (DAPI) overlay. Single-plane confocal images. Arrows point at cCas3-positive cells. Scale bar = 100 μm.

Supplemental Figure 6

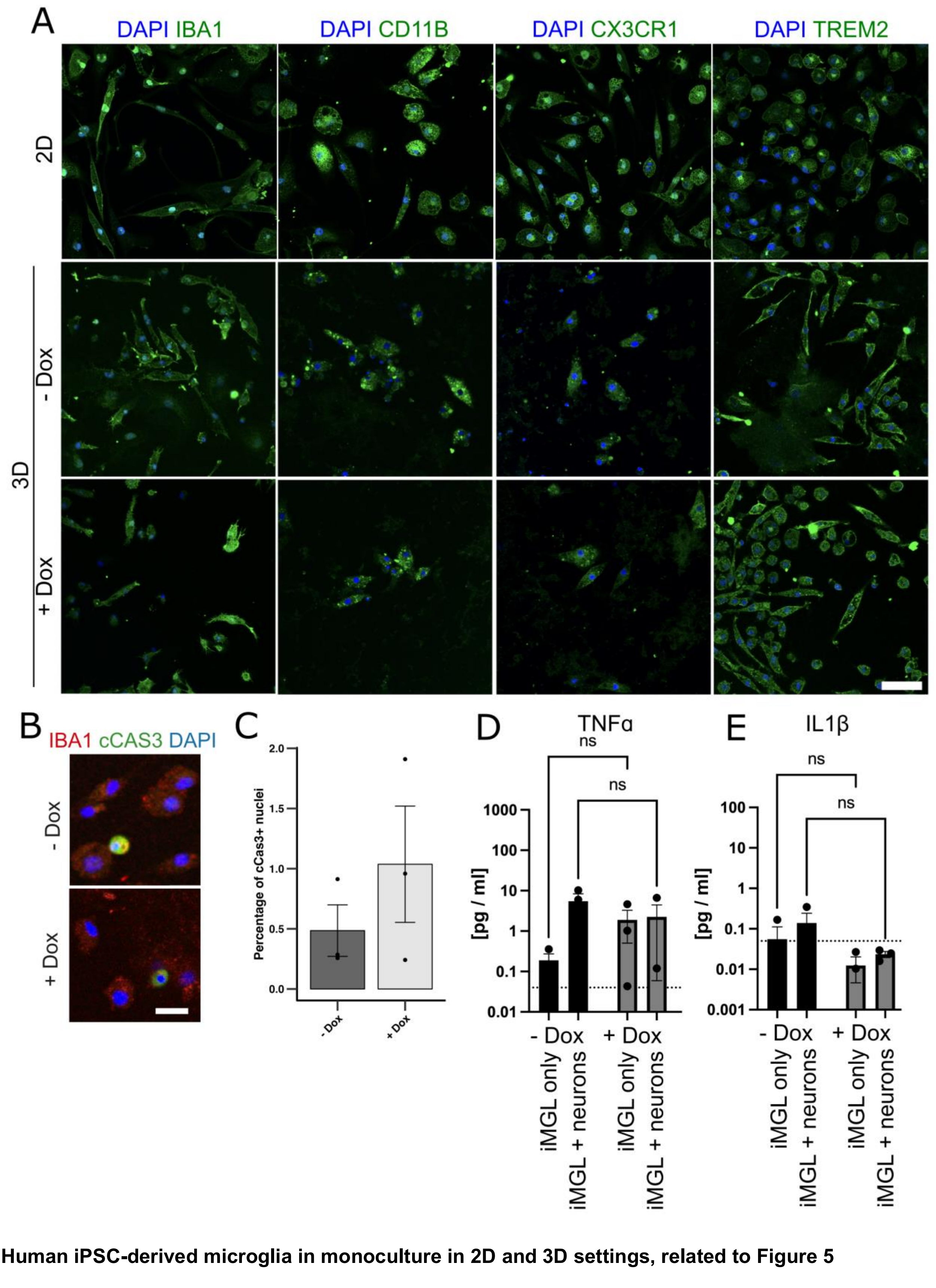

Human iPSC-derived microglia in monoculture in 2D and 3D settings, related to Figure 5

(**A**) Representative confocal images of microglial marker expression after 5 days in 2D or 3D culture. Scale bar = 50 µm. (**B**) Representative confocal images and (**C**) quantification of cCAS-positive cells in the

presence and absence of 1 µg/ml doxycycline (n=3). Scale bar = 25 µm. (**D**) MSD quantification of TNFα secretion into conditioned medium from 3D cultures of iPSdMiGs with or without a 5-day-co-culture with neurons. (**E**) MSD quantification of IL1β secretion into conditioned medium from 3D cultures of iPSdMiGs with or without a 5-day-co-culture with neurons. Kruskal-Wallis test with Nemenyi's post-hoc test. All data presented as mean ± SEM.
